# Identification of the *chaA* and *fwA* Spore Color Genes of *Aspergillus nidulans*

**DOI:** 10.3390/jof10020104

**Published:** 2024-01-26

**Authors:** C. Elizabeth Oakley, Thomas S. Barton, Berl R. Oakley

**Affiliations:** Department of Molecular Biosciences, University of Kansas, 1200 Sunnyside Ave., Lawrence, KS 66045, USA; ceo@ku.edu (C.E.O.); tjayhawk08@ku.edu (T.S.B.J.)

**Keywords:** *Aspergillus nidulans*, spore, conidia, polyketide, pigment

## Abstract

Wild-type *Aspergillus nidulans* asexual spores (conidia) are green due to a pigment that protects the spores against ultraviolet light. The pigment is produced by a biosynthetic pathway, the genes of which are dispersed in the genome. The backbone molecule of the pigment is a polyketide synthesized by a polyketide synthase encoded by the *wA* gene. If *wA* is not functional, the conidia are white. The polyketide is modified by a laccase encoded by the *yA* gene and inactivation of *yA* in an otherwise wild-type background results in yellow spores. Additional spore color mutations have been isolated and mapped to a locus genetically, but the genes that correspond to these loci have not been determined. Spore color markers have been useful historically, and they remain valuable in the molecular genetics era. One can determine if a transforming fragment has been successfully integrated at the *wA* or *yA* locus by simply looking at the color of transformant conidia. The genes of the potentially useful color loci *chaA* (chartreuse conidia) and *fwA* (fawn conidia) have not been identified previously. We chose a set of candidate genes for each locus by comparing the assembled genome with the genetic map. By systematically deleting these candidate genes, we identified a cytochrome P450 gene (AN10028) corresponding to *chaA*. Deletions of this gene result in chartreuse conidia and chartreuse mutations can be complemented in trans by a functional copy of this gene. With *fwA*, we found that the existing fawn mutation, *fwA1*, is a deletion of 2241 base pairs that inactivates three genes. By deleting each of these genes, we determined that *fwA* is AN1088, an EthD domain protein. Deletion of AN1088 results in fawn conidia as expected. Neither deletion of *chaA* nor *fwA* restricts growth and both should be valuable target loci for transformations. Combinations of deletions have allowed us to investigate the epistasis relationships of *wA*, *yA*, *chaA* and *fwA*.

## 1. Introduction

Pigments of conidia (asexual spores) of fungi have a variety of functions such as protecting the conidia against ultraviolet light, ionizing radiation and oxidizing agents, and some play a role in pathogenesis [1]. The conidia of *Aspergillus nidulans* contain a green pigment that has been shown to protect against ultraviolet light [1,2,3] and it may have additional, as yet undiscovered, functions. These pigments are secondary metabolites, compounds that are not strictly required for viability, but confer a selective advantage to the producing organism. Most fungal secondary metabolites are produced by biosynthetic pathways encoded by clusters of genes contiguous in the genome and co-ordinately regulated. Extensive genetic analysis has shown, however, that the genes responsible for synthesis of the conidial pigment in *A. nidulans* are not contiguous but are widely separated in the genome.

**Figure 1:**
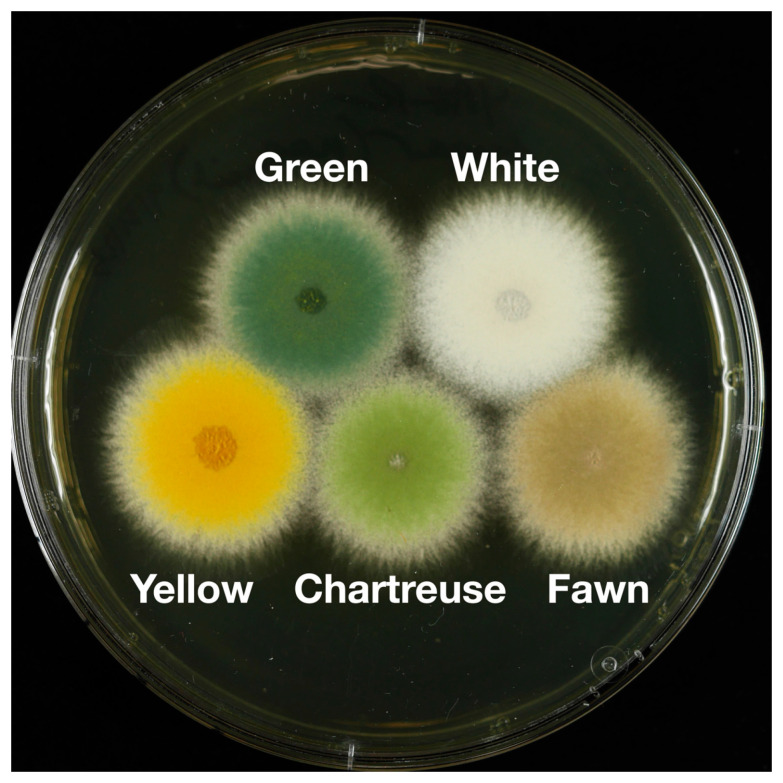
Colonies showing wild-type (Green) and mutant spore colors. Incubation was at 37 °C for 48 h. The medium is YAG + 1 M sucrose with riboflavin to supplement a riboflavin nutritional requirement in two of the strains.

The *wA* gene (AN8209 using the FungiDB.org designation) encodes a polyketide synthase (WA) that synthesizes YWA1, the backbone polyketide of the conidial pigment [4,5]. If this gene is not functional, the conidial pigment is not produced, and the conidia are white (Figure 1). The *yA* (yellow conidia) locus encodes a *p*-diphenol oxidase or laccase (YA) [6,7,8]. If *yA* is dysfunctional in a *wA*+ background, the conidia are yellow, indicating that *yA* is required to modify YWA1, and inactivation of *yA* results in accumulation of the yellow pigment [6,9]. *yA* and *wA* have been very useful genetic markers historically and they are of considerable value in molecular genetics because one can determine if a transforming fragment has been successfully inserted at one of these loci by simple visual inspection of transformants. This can be very useful in heterologous expression experiments, for example [10]. 

The genes for additional historically important and potentially useful color loci have not yet been identified. Chartreuse (*chaA*) (Figure 1) was a spontaneous mutant originally identified by Käfer [11] and fawn (*fwA*) (Figure 1) was a spontaneous mutant originally identified by Clutterbuck [12]. Although these genetic loci have not been matched with particular genes in the assembled genome, they have been mapped genetically. The goals of this project are to identify the genes that correspond to the *chaA* and *fwA* loci, to determine if they are useful target loci for transformations and to use individual, double and triple deletion strains to explore the epistasis relationships among *wA*, *yA*, *chaA* and *fwA*.

The assembled genome and genetic map align well enough to allow us to define regions of the genome from which to choose a set of candidate genes for each locus. With these candidates selected, we used molecular genetic methods to identify the correct gene for each locus. We have determined that the *chaA* gene is a putative cytochrome P450 gene (AN10028 using the FungiDB gene designation; https://fungidb.org/fungidb/app (accessed on 18 January 2024). Deletion of AN10028 results in chartreuse conidia as expected and targeted integration of AN10028 elsewhere in the genome complements a *chaA* mutation, resulting in green colonies. 

We have found that the *fwA* gene is AN1088. It encodes a protein with a putative EthD domain. The original *fwA1* allele removes much of the promoter of AN1088, all of the adjacent gene AN1087 and a substantial portion of an additional gene, AN11841. Inactivation of AN1087 or AN11841 does not result in fawn colonies, but deletion of AN1088 does. With the original *fwA1* allele, repair of the promoter of AN1088 restores the wild-type green spore color. Both *chaA* and *fwA* should be valuable target loci for transformations and deletion of these genes has allowed us to investigate the epistasis relationships of *wA*, *yA*, *chaA* and *fwA*.

## 2. Materials and Methods

### 2.1. Media

YG (5 g/L yeast extract (Fisher Scientific, Pittsburg, PA, USA), 20 g/L D-glucose (Sigma-Aldrich, St. Louis, MO, USA) and 800 μL of a trace element solution [13]) was used as a complete medium. For strains carrying *pyrG* mutations it was supplemented with 10 mM uridine (Fisher Scientific, Pittsburg, PA, USA) and 8.9 mM uracil (Beantown Chemicals, Hudson, NH, USA). Because some batches of yeast extract are deficient in riboflavin, we added riboflavin (Sigma-Aldrich, St. Louis, MO, USA) to a final concentration of 2.5 μg/mL. Solid complete medium was YAG (YG + 15 g/L agar (Technova, Holister, CA, USA)). Solid minimal medium was 10 g/L D-glucose, 15 g/L agar, 2 mL/L of a 26% *w*/*v* MgSO_4_∙7H_2_O (Avantor, Radnor, PA, USA) solution, 800 μL/L trace element solution [13], 6 g/L NaNO_3_ (Fisher Scientific, Pittsburg, PA, USA), 0.52 g/L KCl (Sigma-Aldrich, St. Louis, MO, USA) and 1.52 g/L KH_2_PO_4_ (Sigma-Aldrich, St. Louis, MO, USA) (all values are final concentrations). The latter three salts were from a 10X stock salts solution adjusted to pH 6.0–6.5 with saturated KOH (Sigma-Aldrich, St. Louis, MO, USA), autoclaved separately from the other components, cooled to 60 °C and mixed with the solution containing the other components (100 mL + 900 mL) to give the stated final concentrations.

### 2.2. Strains

Strains used or created in this work are shown in Table 1.

### 2.3. Molecular Genetic Methods

DNA fragments to be used for fusion PCR were amplified using Q5 DNA polymerase (New England Biolabs (Ipswich, MA, USA)) with an annealing temperature of 65 °C (Tm + 5). PCR reactions were carried out with a TC-32 Mini Thermal Cycler (Benchmark, Scientific, Sayreville, NJ, USA). Fragments were purified using a Qiaquick PCR purification kit (Qiagen, Germantown, MD, USA). Fusion PCR was carried out using the method of Szewczyk et al. [13] as modified by Oakley et al. [14] using Q5 DNA polymerase (New England Biolabs). The annealing temperature was 65 °C (Tm + 5) and the volumes of the reactions were 50 μL. Fusion PCR products were purified using Monarch PCR & DNA Cleanup Kits (New England Biolabs) and eluted in a volume of 20 μL of elution buffer. Conidial DNA minipreps were carried out using the method of Edgerton-Morgan and Oakley [15]. Diagnostic PCR on minipreps was carried out using OneTaq HS Quick-load 2X Master Mix (New England Biolabs) at an annealing temperature of 60 °C.

To clone the *fwA1* allele, Q5 polymerase was used to amplify the relevant fragment from cesium-chloride-purified DNA from strain G191, which carries *fwA1*. The fragment was purified with a Monarch PCR & DNA cleanup kit, eluted in 20 μL of elution buffer and sequenced by Eurofins (Louisville, KY, USA). All primers used for this work were synthesized by Integrated DNA Technologies, Inc., (Coralville, IA, USA) are listed in the Supplementary Materials.

### 2.4. Transformation Procedure

Transformations were carried out using an updated version of previously published procedures [13,14]. A total of 1 × 10^8^ conidia were inoculated into 20 mL of complete medium in a 50 mL Erlenmeyer flask and incubated at 30 °C overnight, shaking at 140 rpm in a New Brunswick G76D gyrotory shaker (New Brunswick Scientific, Edison, NJ, USA). Hyphae were harvested by filtering through sterile Miracloth (Calbiochem, San Diego, CA, USA) and washed with ≥40 mL of sterile distilled water followed by ≥5 mL of fresh culture medium. The hyphae were then resuspended in 8 mL of fresh complete medium in a fresh, sterile 50 mL Erlenmeyer flask, and 8 mL of 2X protoplasting solution [13,14] (sterilized by filtration through a MilliporeSigma Stericup Quick Release-GV Sterile Vacuum Bottle Top Filtration System (Fisher Scientific, Pittsburg, PA, USA) was added. VinoTaste Pro (Novozymes, Bagsvaerd, Denmark) was the protoplasting enzyme in the solution and it was used at a final concentration of 200 mg/mL. The flask was incubated at 30 °C, shaking at 110 rpm in a New Brunswick G76D gyrotory shaker. Hyphae were repeatedly drawn into and expelled from a sterile transfer pipet at 15 min intervals to break up any hyphal clumps. Protoplasting was monitored by phase contrast microscopy and, when protoplasting was substantially complete (usually about 2 h), the flask was placed in an ice bucket with gentle shaking for 10 min. The protoplast suspension was layered over 15 mL of 1.2 M sucrose (Fisher Scientific, Pittsburg, PA, USA) in a sterile 50 mL centrifuge tube and centrifuged at 2800× *g* for 10 min at 4 °C. The sucrose solution and centrifuge tube were chilled on ice before use and slow acceleration and deceleration settings were used during centrifugation to prevent mixing of the solutions. The interface was collected and transferred to a 15 mL sterile centrifuge tube and a volume of 0.6 M KCl equal to or greater than the volume of the collected interface was added and the suspension was mixed well. The protoplasts were pelleted at 2400× *g* for 10 min at 20 °C. The supernatant was removed, 1 mL of 0.6 M KCl was added and the protoplasts were suspended by drawing the liquid into and expelling it from a micropipette tip. The protoplasts were washed three times by pelleting at 3200× *g* in a swing-out microcentrifuge and resuspending in 1 mL of 0.6 M KCl. The protoplasts were then resuspended in 1 mL of 50 mM CaCl_2_ (Fisher Scientific, Pittsburg, PA, USA), 0.6 M KCl solution. Ten microliters of this suspension was examined by phase contrast microscopy to assess protoplast morphology and approximate number. The protoplasts were pelleted by centrifugation at 3200× *g* and resuspended in a volume of 50 mM CaCl_2_, 0.6 M KCl solution appropriate for the number of transformations to be carried out. Typically, we have enough protoplasts for 10 transformations yielding ≥20 transformants but, if we are carrying out fewer transformations, we use more protoplasts per transformation and obtain correspondingly more transformants. A total of 40 μL of protoplast suspension was used per transformation. DNA fragments or plasmid were added to ≤6 μL of TE buffer (10 mM Tris (Sigma-Alcrich, St. Lous, MO, USA), 1 mM EDTA (Fisher Scientific, Pittsburg, PA, USA), pH 8.0), 20 μL of freshly filtered room temperature PEG solution [13] was added and the suspension was mixed by vortexing. The suspension was incubated in an ice water bath for 25 min, and 400 μL of freshly filtered room temperature PEG solution was then added and the suspension was mixed by drawing it into and expelling it from the micropipette tip used to add the PEG solution. After incubation at room temperature for 30 min, 100 μL aliquots were plated directly onto selective media plates.

## 3. Results

### 3.1. Identification of the chaA Gene

*ChaA* maps between *panA* (AN0205) and *ivoB* (AN0231) on linkage group VIII (Supplementary Materials Figure S1) https://www.fgsc.net/Aspergillus/gene_list/viiicontigs.html (accessed on 10 January 2024). In this region, we found three genes with predicted enzymatic activities consistent with a role in pigment modification, AN0215 and AN0216, both putatively encoding oxyreductases, and AN10028, which putatively encodes a cytochrome P450. The genomic position of AN10028 notably correlated precisely with the genetic mapping data.

**Figure 2:**
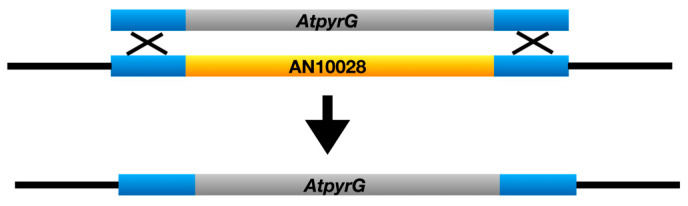
Deletion of AN10028 (yellow) by replacement with *AtpyrG* (grey). A fragment carrying the *AtpyrG* gene and sequences that flank AN10028 (blue) was created by fusion PCR. Transformation and resulting homologous recombination in the flanking regions results in replacement of AN10028 with *AtpyrG*.

**Figure 3:**
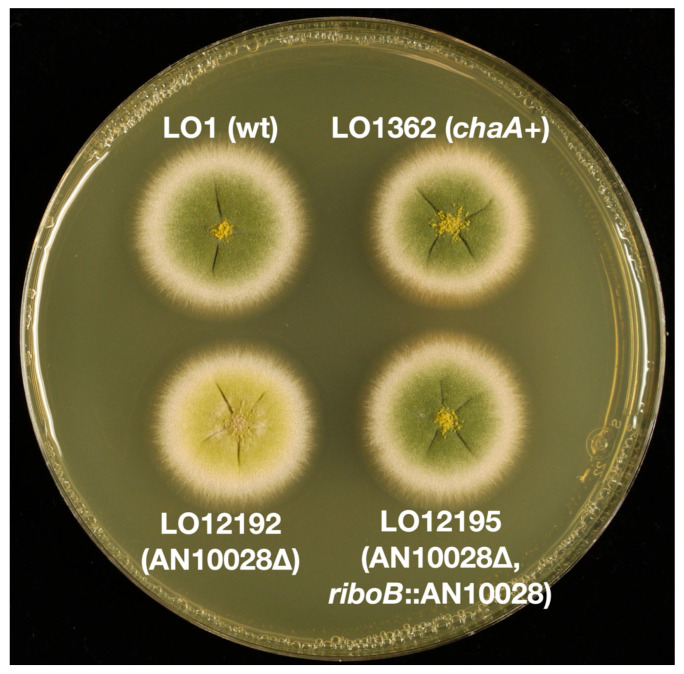
Deletion of AN10028 results in chartreuse colonies. LO1 is a wild-type strain that has green conidia. LO1362 is the parental strain for LO12192. It carries three selectable markers and the *nkuA* deletion, but it is wild-type for color markers. LO12192 is LO1362 transformed with the fragment shown in Figure 2 to delete AN10028. This deletion resulted in the chartreuse spore color. Insertion of a wild-type copy of AN10028 at the *riboB* locus of LO12192 resulted in strain LO12195, in which the wild-type green color is restored. Growth was for 48 h at 37 °C on YAG with uridine, uracil and riboflavin added to supplement nutritional requirements. Note that colonies grown on YAG with sucrose are smoother than colonies grown on YAG alone such as these (see Figure 1 for comparison).

Deletions of the *wA* and *yA* genes give white and yellow conidia respectively. The conidial colors are thus due to the loss of function of the *wA* and *yA* genes. We, therefore, hypothesized that deleting the *chaA* gene in a green conidia background might result in the chartreuse conidia phenotype. We used fusion PCR to create transforming fragments to delete each candidate gene by replacing it with the *Aspergillus terreus pyrG* gene (*AtpyrG*) (Figure 2). We transformed strain LO1362 (which is synonymous with TN02A7 [16] and FGSC A1145) that carries a deletion of the *nkuA* gene, which greatly reduces off-target integration of transforming fragments. When AN0215 and AN0216 were deleted, all transformants were green (e.g., Supplementary Materials Figure S2) but with the AN10028 deletion the great majority of transformants (117/127) were chartreuse (Figure 3), with a small minority of green transformants presumably due to off-target insertions of the transforming fragment. We carried out multiple diagnostic PCR reactions on five chartreuse transformants and verified that AN10028 was deleted in each case (Supplementary Materials Figures S3–S6). These data indicate very strongly that AN10028 is the *chaA* gene. 

It is also worth noting that *AtpyrG* fully complements *pyrG89* when integrated at the *chaA* locus. A concern that is sometimes raised with expression of genes integrated at color marker loci is that, since the products of color marker genes function during conidiation, there could be chromatin-level position effects repressing expression of the locus at other times in the life cycle. We have found good expression during vegetative growth of genes inserted at other conidial color marker loci, however (e.g., [10] and additional unpublished data), and the fact that *AtpyrG* integrated at the *chaA* locus fully complements *pyrG89* during vegetative growth suggests that there is little or no repression of the locus. 

**Figure 4:**
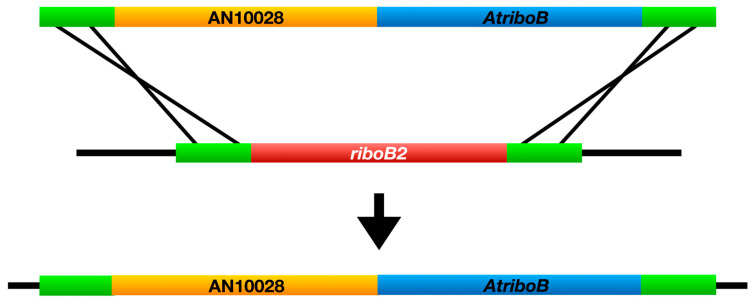
Insertion of a full-length copy of AN10028 at the *riboB* locus. *RiboB2* is a mutant allele that does not support growth in the absence of riboflavin. *AtriboB* is the homolog of *riboB* from *Aspergillus terreus*. It functions in *A. nidulans* and complements *riboB2*. A full-length copy of AN10028 was amplified from wild-type *A. nidulans* DNA and fused to *AtriboB* and sequences that flank the *riboB* gene. Transformation resulted in AN10028 being inserted at the *riboB* locus along with *AtriboB*.

To confirm that AN10028 is indeed the *chaA* gene, we attempted to complement the *chaA* deletion in trans with AN10028. We used fusion PCR to create a transforming fragment to land a full-length copy of AN10028 at the *riboB* locus (Figure 3 and Figure 4). The transforming molecule consisted of a 967 bp 5’ *riboB* flanking sequence, a 2366 bp sequence containing 559 bp of 5’ AN10028 untranslated region (UTR) (including the AN10028 promoter), the AN10028 coding region, 938 bp of *riboB* 3’ flanking sequence, and a 2008 bp fragment of *A. terreus* DNA that carries the *A. terreus riboB* gene (*AtriboB*). The *AtriboB* fragment included a 467 bp 5’ UTR that extends from the stop codon of the adjacent gene (thereby providing a functional 3’ UTR for AN10028), and it also included a 199 bp *AtriboB* 3’ UTR. The *chaA* deletants carry a dysfunctional, mutant *riboB* allele, *riboB2*. This allele is complemented by *AtriboB*, but the nucleotide sequences of the two genes are sufficiently different that the *AtriboB* sequence does not direct integration of the transforming fragment. Rather, the *riboB* flanking sequences on the transforming fragment direct integration by homologous recombination such that the transforming fragment replaces the *riboB2* allele with *AtriboB* and AN10028. Transformants can, thus, be selected on the basis of riboflavin prototrophy and, if AN10028 is, indeed, *chaA*, the great majority of the transformants should be green. We transformed two *chaA* deletant strains, LO12192 and LO12193. With LO12192, 139/140 transformants were green and, with LO12193, 104/105 transformants were green. Multiple diagnostic PCR reactions on six green transformants confirmed that the transforming fragment had integrated by homologous recombination at the *riboB* locus as expected (Supplementary Materials Figures S7–S9). These data confirm that AN10028 is the *chaA* gene.

### 3.2. Identification of the fwA Gene

**Figure 5:**
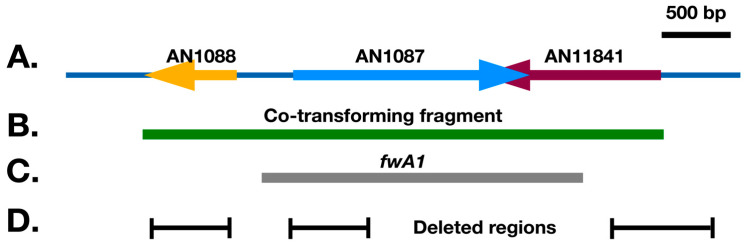
(**A**) The region containing AN1088, AN1087 and AN11841. Analysis of RNA sequences (beyond the scope of this manuscript) reveal that the transcripts of AN1087 and AN11841 overlap substantially, although their likely stop codons are 19 bp apart such that their coding sequences do not overlap. (**B**) The green bar is the PCR-amplified fragment that complements *fwA1* in co-transformation experiments resulting in green transformants. (**C**) The grey bar shows the region deleted in the *fwA1* mutation. (**D**) Regions deleted to inactivate AN1088, AN1087 and AN11841. The region is drawn to scale. Bar = 500 bp.

Available mapping data (https://www.fgsc.net/Aspergillus/gene_list/viiimap.html (accessed on 10 January 2024)) do not pinpoint the position of *fwA* as precisely as that of *chaA*. *FwA* is 12 centimorgans centromere proximal to *veA* (AN1052) on linkage group VIII and about 36 centimorgans centromere distal to *qutD* (AN1138) (Supplementary Materials Figure S10). In this region, we found four *fwA* candidates, AN1059 (putatively encoding a carnitine acetyltransferase), AN1063 (putative NADH dehydrogenase), AN1087 (putative cytochrome P450) and AN10167 (putative role in oxidation and reduction). Note, however, that AN1087 is between two genes that could plausibly have roles in pigment formation (Figure 5). One, AN1088, is annotated as encoding a protein of unknown function that contains an EthD domain. Some EthD domain proteins have been shown to be involved in pigment formation (see discussion below). The other is AN11841, which is predicted to encode an acetyl transferase. 

To identify *fwA*, we used a different strategy from that used to identify *chaA*, a co-transformation strategy that we have used previously [17]. In strains in which the nonhomologous end-joining system is functional, if one transforms with two DNA molecules, one carrying a selectable marker and one without a selectable marker, some transformants selected for the selectable marker also are transformed with the non-selectable DNA fragment. In this instance, if we transform a strain carrying a fawn mutation, *fwA1,* and a pyrimidine auxotrophy mutation, *pyrG89,* with a wild-type *pyrG* gene and a fragment carrying a wild-type *fwA* allele, a fraction of the *pyr^+^* transformants should be co-transformed with *fwA* and, thus, have green conidia. We, consequently, PCR amplified, from genomic DNA of the wild-type strain LO1, fragments carrying full-length copies of each of the four candidate genes and co-transformed each fragment along with pPL6, a plasmid carrying the wild-type *pyrG* gene, into strain G191, which carries *fwA1* and *pyrG89* [18,19]. Transformants with green conidia were only seen with the fragment carrying AN1087 as the co-transforming fragment (Table 2). This fragment extended from 981 bp 5’ to the putative AN1087 start codon to 995 bp 3’ to the putative AN1087 stop codon.

If AN1087 is the *fwA* gene, transformation with a fragment designed to delete this gene is predicted to result in transformants with fawn conidia. When we transformed with a fragment designed to delete AN1087 in strain LO1362 by replacing it with *AtpyrG* as a selectable marker (similar to Figure 2), however, all transformants had green conidia. This created an apparent contradiction, with a fragment containing wild-type AN1087 being able to complement the *fwA* mutation but deleting AN1087, not resulting in fawn conidia. We consequently re-examined the region of linkage group VIII that contains AN1087 (Figure 5). AN1087 and AN11841 are extremely close to each other. They are transcribed toward each other, and the 3’ untranslated regions of their mRNAs overlap substantially (transcription data available at FungiDB.org and our unpublished data). Because they are so close, the AN1087-containing fragment that complemented *fwA1* not only contained all of AN1087 but also all of the coding sequence of AN11841. AN1087 and AN1088 are divergently transcribed and their coding regions are about 400 bp apart. The fragment that complemented *fwA1* covered the entire AN1088 promoter and coding sequence. The fragment that complemented *fwA1* could, thus, have complemented mutations in AN1087, AN1088 or AN11841.

In addition, we obtained anomalous PCR fragment lengths when we amplified the region in the *fwA1* mutant strain G191, which suggested that there might be a deletion of slightly more than 2 kb in the region. This result was subsequently repeated with other strains carrying *fwA1*. We consequently amplified the region from G191 and sequenced it. Sequencing revealed that *fwA1* is a 2241 bp deletion that starts 141 bp upstream of the AN1088 start codon, removes all of AN1087 and truncates AN11841, leaving only the n-terminal 137 amino acids of AN11841 intact (Supplementary Materials Figure S11). *FwA1* thus removes AN1087, leaves only a portion of AN11841 that is likely dysfunctional and possibly removes much of the promoter of AN1088. The failure of deletion of AN1087 to cause a fawn phenotype in combination with the fact that *fwA1* likely inactivates all three genes suggested that *fwA* might be AN1088 or AN11841. To determine if this is the case, we inactivated these genes. We also inactivated AN1087 again using a slightly different strategy from the one we used previously to confirm that it is not *fwA*. 

Since AN1087 and AN11841 are close together and have overlapping transcripts, we wanted to be certain to inactivate each gene individually and not inadvertently inactivate both. AN1088 is also very close to AN1087, and they are divergently transcribed. In inactivating AN1088, we deleted the coding region of AN1088 but none of the promoter region between AN1087 and AN1088, thus leaving the AN1087 coding region and promoter intact (Figure 5D). To be sure that when we inactivated AN1087 we did not inactivate AN1088, we deleted a region from 100 bp 5’ to the start codon of AN1087 to 400 bp 3’ to the start codon. This left the AN1088 coding region and about 300 bp of its promoter region intact. For AN11841, we deleted a region from 300 bp 5’ to the start codon to 400 bp 3’ to the start codon. We, thus, removed a portion of the promoter and a substantial portion of the coding sequence including the start codon. We inactivated each gene in a green strain, LO1362, as well as a chartreuse strain, LO12192.

**Figure 6:**
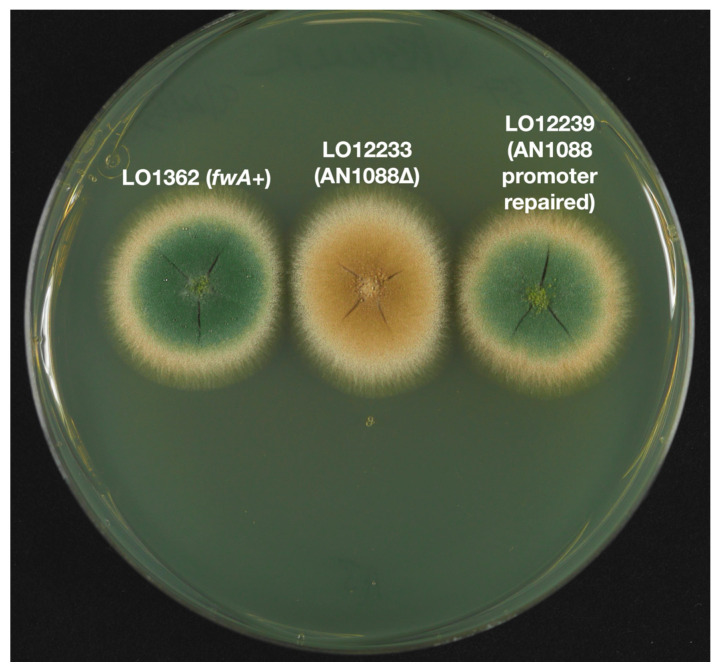
Deletion and promoter repair of AN1088. Deletion of AN1088 in the green strain LO1362 results in fawn conidia (LO12233). Repair of the promoter of AN1088 in a *fwA1* strain (LO2460) results in green conidia (LO12239). Growth was for 48 h at 37 °C on YAG with uridine, uracil and riboflavin to supplement nutritional requirements.

**Figure 7:**
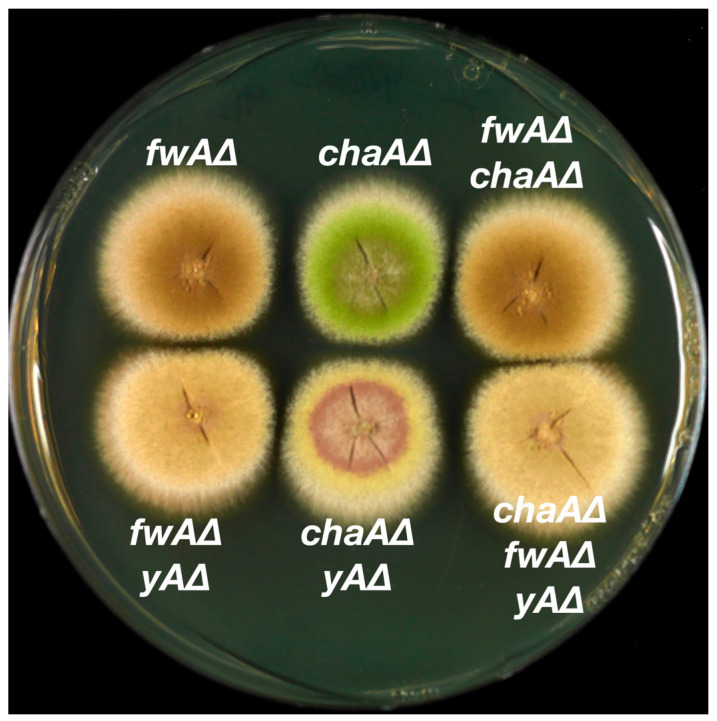
Colonies of strains carrying single, double and triple deletions of *fwA*, *chaA* and *yA*. Top row left, strain LO12233 carries a deletion of *fwA* made by replacing *fwA* (AN1088) with *AtpyrG*. Top row center, strain LO12192 carries a deletion of *chaA* made by replacing *chaA* (AN10028) with *AtpyrG*. Top row right, strain LO12236 carries a deletion of *fwA* made by replacing it with *AtriboB* and a deletion of *chaA* made by replacing it with *AtpyrG*. Bottom row left, strain LO12248 carries a deletion of *fwA* made by replacing it with *AtpyrG* and a deletion of *yA* (AN6635) made by replacing it with *AtriboB*. Bottom row center, strain LO12245 carries a deletion of *chaA* made by replacing it with *AtpyrG* and a deletion of *yA* made by replacing it with *AtriboB*. Bottom row right, strain LO12251 carries deletions of *chaA* made by replacing it with *AtpyrG*, *fwA* made by replacing it with *AtriboB* and *yA* made by replacing it with the *Aspergillus fumigatus pyroA* gene (*AfpyroA*). Growth was for 48 h at 37 °C on YAG supplemented with riboflavin.

Results are shown in Table 3. Targeted inactivation of neither AN1087 nor AN11841 resulted in fawn transformants. However, transformation with a construct designed to remove the coding sequence of AN1088 resulted in a very high frequency of fawn transformants, which we have confirmed by diagnostic PCR to be AN1088 deletants (Supplementary Materials Figures S12–S14). One is shown in Figure 6. These data indicate strongly that AN1088 is the *fwA* gene. Note also that deletion of AN1088 in the chartreuse strain LO12192 resulted in fawn conidia (Figure 7). This demonstrates that the deletion of *fw* is epistatic to the deletion of *chaA* (see discussion below). 

**Figure 8:**
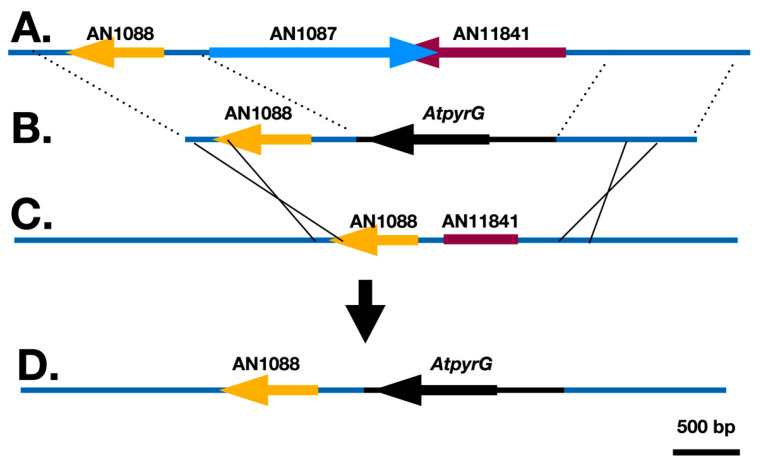
Repair of the promoter of AN1088. (**A**) shows the wild-type *fwA* region. (**B**) shows a transforming fragment made by amplifying a 1240 bp fragment extending from 338 bp upstream of the AN1088 start codon into the 3’ UTR of AN1088. This fragment was fused to a fragment carrying *AtpyrG* and a 1022 bp fragment upstream of the AN11841. This fragment was transformed into strain LO2647 that carries the selectable marker *pyrG*89 as well as the *fwA1* mutation shown in (**C**). Note that *fwA1* eliminates all of AN1087, much of the 5’ UTR of AN1088 and truncates the coding region of AN11841. The result of homologous recombination is that AN1087 and AN11841 are deleted completely, whereas the promoter of AN1088 is repaired (**D**). A parallel experiment was carried out in which a 2186 bp fragment containing the *AfpyroA* gene was used as the selectable marker instead of *AtpyrG* and the recipient strain was LO2460, which carries *pyroA4* as well as *fwA1*.

To confirm that AN1088 is *fwA*, we created constructs to repair the promoter of AN1088 in strains carrying *fwA1* (Figure 8). One construct carried *AtpyrG* as a selectable marker. This construct was transformed into strain LO2647 which carries the *pyrG89* mutation as well as *fwA1*. The second construct carried the *Aspergillus fumigatus pyroA* gene (*AfpyroA*) and it was transformed into LO2460, which carries *pyroA4* as well as *fwA1*. If AN1088 is, indeed, the *fwA* gene, transformation with these constructs should result in restoration of AN1088 function and the great majority of transformants should have green conidia. Transformation with these constructs resulted in high frequencies of green transformants as predicted. With LO2460, 332 transformants were scored and all were green (one is shown in Figure 6). With LO2647, the transformation frequency was so high that it was not possible to determine the exact number of transformants but, of 800 scored, all were green (diagnostic PCR confirmation in Supplementary Materials Figures S15 and S16). These transformations also deleted AN1087 and AN11841, demonstrating that neither of these genes are essential for viability. The deletion and restoration of function data, in combination, allow us to conclude unambiguously that AN1088 is *fwA*. It is noteworthy that, in the original fawn mutant, *fwA1*, three genes are inactivated (AN1087, AN1088 and AN11841). While our data reveal that none of these genes are essential for viability, the *fwA1* allele cannot be considered to be solely a color marker because inactivation of AN1087 and AN11841 might confer phenotypes other than conidial color alteration under some conditions.

AN1088 is a previously uncharacterized gene. Its most notable feature is that it contains an EthD domain. The EthD family was originally described in bacteria. The first described member of the family was the EthD gene of *Rhodococcus ruber* where it is involved in the degrading of ethyl *tert*-butyl ether [20]. EthD proteins have subsequently been described in other bacteria as well as fungi. In fungi, EthD proteins have notably been found in gene clusters involved in synthesis of pigments or colored compounds such as anthraquinones and the structurally related xanthones [21,22,23,24]. Griffiths et al. [24] have demonstrated, moreover, that the products of the *mdpH* gene of *A. nidulans,* which is involved in xanthone biosynthesis and carries an EthD domain, and the *claH* gene of *Cladosporium fulvum*, which is involved in biosynthesis of anthraquinones and also carries an EthD domain, are decarboxylases. 

### 3.3. Epistasis Relationships among Conidial Color Markers

The fact that the *wA*, *chaA*, *yA* and *fwA* genes have now been identified allows us to create clean deletions and combinations of deletions and/or mutations in the same genetic background. This facilitates examining the epistasis relationships among the genes without the complications of different genetic backgrounds or, in the case of *fwA1*, the fact that the original mutation inactivated three genes. Epistasis relationships give clues as to the order in which the genes function in the conidial color biosynthetic pathway. They also have a practical value in that it is often useful to replace color marker genes with transforming genes, because the correct integrations are immediately apparent from the color of the transformed colonies. By creating double and triple deletions, we can determine which combinations of gene replacements of color markers can be identified from the colony color. We, therefore, created single, double and triple deletions of *yA*, *chaA* and *fwA* by replacing each gene with a selectable marker. The colors of the transformants were extremely consistent and individual examples are shown in Figure 7.

It has long been known that the *wA* polyketide synthase synthesizes the backbone polyketide for the conidial pigment. In the absence of *wA* function, the conidial pigment is not produced and the conidia are white. In other words, *wA* is epistatic to all the other color marker genes. The *fwAΔ* deletion is indistinguishable from the *fwA∆*, *chaA∆* double deletion (Figure 7). *fwA* is thus epistatic to *chaA*. The *fwA∆*, *yA∆* double mutant is fawn in color, but it is slightly paler than the *fwA∆* single mutant. These two deletions, thus, combine to create a novel (albeit subtle) phenotype. The *fwA∆*, *chaA∆*, *yA∆* triple mutant is indistinguishable from the *fwA∆*, *yA∆* double mutant, consistent with *fwA∆* being epistatic to *chaA∆* but creating a novel phenotype with *yA∆*. Finally, the *yA∆*, *chaA∆* double mutant has a novel phenotype. It is a paler yellow than *yA∆* strains and colonies develop a brown ring or disk around the center of the colony that becomes apparent as the colony grows.

## 4. Discussion

We have successfully identified the genes that correspond to the *chaA* (AN10028) and *fwA* (AN1088) color marker loci of *A. nidulans*. ChaA is a predicted cytochrome P450 and FwA is an EthD protein. Both are predicted to have enzymatic functions rather than regulatory functions. It is, thus, likely that they chemically modify the spore color pigment at some stage in its synthesis. It follows that *fwA* and *chaA* deletions are likely to be helpful in deciphering the biosynthetic pathway for the conidial pigment. Epistasis relationships give clues as to the biosynthetic pathway. *wA* is epistatic to all spore color loci. YWA1, the compound synthesized by the WA polyketide synthase is the backbone polyketide for the conidial pigment molecule [4,5]. If *yA* is not functional and *fwA* is functional, a bright yellow pigment accumulates. If, however, *fwA* is deleted in a background in which *yA* is not functional, spores are a pale fawn in color. This result is inconsistent with *yA* being upstream of *fwA* because, if it were, the spores of the double mutant would be the bright yellow of *yA* single mutants. The results are more consistent with both *fwA* and *yA* modifying YWA1. If *fwA* is functional, the *chaA*, *yA* double mutant has a novel phenotype with pale yellow conidia and an accumulation of a brown pigment in the centers of colonies as they grow. However, *fwA* is epistatic to *chaA*. The *fwA*, *chaA* double deletant is identical to the *fwA* single deletant and the *fwA*, *yA*, *chaA* triple deletion is identical to the *fwA*, *yA* double deletion. One feasible explanation for these data is that FwA, the EthD protein encoded by *fwA*, and YA, the laccase encoded by *yA*, both independently modify YWA1 and the cytochrome P450 encoded by *chaA* modifies the compound produced when FwA modifies YWA1. 

Finally, *fwA* and *chaA* are likely to be very useful target loci for landing genes. Transformants in which these genes are replaced with other genes are readily identifiable from colony color and deletions of these loci do not inhibit growth. In addition, if desired, one can carry out serial transformations at these color loci. For example, a green strain can be transformed to chartreuse, chartreuse can be transformed to fawn or yellow and fawn or yellow can be transformed to white.

## Figures and Tables

**Table 1:** Strains used in this work.

Strain	Genotype	Notes
G191	*pyrG89*, *pabaA1*, *fwA1*, *uaY9*	From G. Turner via C. F. Roberts
LO1	*A. nidulans* wt, no nutritional requirements	
LO1362	*pyrG89*, *pyroA4*, *riboB2*, *nkuA::argB*	=TN02A7 and FGSC A1145
LO2460	*mad2::AfpyrG*, *pyroA4*, *pabaA1*, *fwA1*, *nkuA::argB* (may carry *pyrG89*)	
LO2647	*pyrG89*, *fwA1*, *nkuA::argB*	
LO12192	*chaA::AtpyrG*, *pyrG89*, *pyroA4*, *riboB2*, *nkuA::argB*	
LO12193	*chaA::AtpyrG*, *pyrG89*, *pyroA4*, *riboB2*, *nkuA::argB*	
LO12195	*riboB2::chaA-AtriboB*, *chaA::AtpyrG pyrG89*, *pyroA4*, *riboB2*, *nkuA::argB*	
LO12233	*fwA::AtpyrG*, *pyrG89*, *pyroA4*, *riboB2*, *nkuA::argB*	
LO12236	*fwA::AtriboB*, *chaA::AtpyrG pyrG89*, *pyroA4*, *riboB2*, *nkuA::argB*	
LO12239	*fwA*(p)-*AfpyroA*, *mad2::pyrG*, *pyroA4*, *pabaA1*, *fwA1*, *nkuA::argB*	Promoter of *fwA* repaired
LO12245	*yA::AtriboB*, *chaA::AtpyrG pyrG89*, *pyroA4*, *riboB2*, *nkuA::argB*	
LO12248	*yA::AtriboB*, *fwA::AtpyrG*, *pyrG89*, *pyroA4*, *riboB2*, *nkuA::argB*	
LO12251	*yA::AfpyroA*, *fwA::AtriboB*, *chaA::AtpyrG pyrG89*, *pyroA4*, *riboB2*, *nkuA::argB*	
R21	*pabaA1*, *yA2*	From C. F. Roberts
R153	*pyroA4*, *wA3*	From C. F. Roberts

**Table 2:** Green co-transformants in a fawn strain. The fawn strain G191 was co-transformed with PCR-amplified fragments containing the genes shown as well as plasmid pPL6 that carries the wild-type *pyrG* allele. Below each gene the numbers of fawn and green transformants are listed. Thus, when the fragment containing AN1059 was co-transformed with pPL6, 37 transformants were obtained and all were fawn. Only the fragment containing AN1087 produced green transformants.

Gene	AN1059		AN1063		AN1087		AN10167	
	Fawn	Green	Fawn	Green	Fawn	Green	Fawn	Green
	37	0	53	0	27	12	61	0

**Table 3:** Fawn transformants obtained in transformations designed to delete the three genes in the AN1088 region. Fw = transformants with fawn conidia, gr = transformants with green conidia, cha = transformants with chartreuse conidia. LO1362 is a strain with green conidia. Deletion of the *fwA* gene should result in the transformants having fawn conidia. LO12192 is a chartreuse strain. If fawn is epistatic to chartreuse, *fwA* deletants should have fawn conidia. Deletion of AN1088 resulted in large numbers of fawn conidia, whereas deletion of AN1087 or AN11841 did not. The single fawn transformant with the AN1087 deletion is presumably a contaminant or anomalous integration and the two chartreuse transformants in the AN1088 deletion transformants are presumably off-target integrations.

Gene Deleted	AN1087				AN1088				AN11841			
Strain	LO1362		LO12192		LO1362		LO12192		LO1362		LO12192	
	fw	gr	fw	cha	fw	gr	fw	cha	fw	gr	fw	cha
	1	141	0	109	216	0	280	2	0	197	0	221

## Data Availability

Data are contained within the article and Supplementary Materials.

## Supplementary Materials

The following supporting information can be downloaded at: https://www.mdpi.com/article/10.3390/jof10020104/s1. List of Primers Used in this Work. Supplemental Figure S1. A map of the region around *chaA*. Supplemental Figure S2. Phenotypes of deletions of *chaA* candidate genes. Supplemental Figure S3. Schematic diagram of diagnostic PCR for deletion of *chaA*. Supplemental Figure S4. Diagnostic PCR of *chaA* deletion transformants using outside primers. Supplemental Figure S5. Secondary diagnostic PCR of putative deletions of AN10028. Supplemental Figure S6. Tertiary diagnostic PCR of putative deletions of AN10028. Supplemental Figure S7. Schematic diagram of diagnostic PCR for restoration of *chaA*. Supplemental Figure S8. Primary PCR verification of the restoration of *chaA*. Supplemental Figure S9. Secondary PCR verification of the restoration of *chaA*. Supplemental Figure S10. A genetic map of the *fwA* region of linkage group VIII. Supplemental Figure S11. The *fwA* region containing three genes, AN1088 (*fwA*), AN1087 and AN11841. Supplemental Figure S12. Schematic diagram of diagnostic PCR to verify the deletion of *fwA*. Supplemental Figure S13. Primary PCR verification of the deletion of *fwA* (AN1088). Supplemental Figure S14. Secondary and tertiary verifications of AN1088 (putative *fwA*) deletions. Supplemental Figure S15. Schematic of diagnostic PCR of AN1088 promoter restoration. Supplemental Figure S16. Diagnostic PCR of AN1088 promoter repair transformants. Reference [25] is cited in Supplementary Materials.
