# Supplementary material for: Identification of the chaA and fwA Spore Color Genes of Aspergillus nidulans
Source: J Fungi (Basel). 2024 Jan 26;10(2):104. doi: 10.3390/jof10020104 (PMC10890192; doi:10.3390/jof10020104)
Supplement: Supplementary file 1 [file jof-10-00104-s001.zip › jof-2812620-supplementary.pdf]

List of Primers used in this work

Note: Lower case sequences are "tails" added to facilitate fusion PCR

Primers for flanks to delete AN0215

Primers for 5' flank:
F, for diagnostic PCR: TTACTGCGAGACAGAGTTCG
F, for fragment amplification: CTATCACGGCGCACGAGC
F, for fusion PCR: AGCTACAATGATCGAGGAATC
R, for fragment amplification: cgaagagggtgaagagcattgCTGTACTGTTTCCCGGGTC

Primers for 3' flank:
F, for fragment amplification: agtgcctcctctcagacagTGTGCGGCATAGTTCATATTG
R, for fusion PCR: ACACTACTAAGCGCAGTTGC
R, for fragment amplification: GATGATGCTATATCTCGCAAG
R, for diagnostic PCR: TGGATTGGATGCTTAACCGG

Primers for flanks to delete AN0216

Primers for 5' flank:
F, for diagnostic PCR: ATTGTCCTAGGCAGGTCTTG
F, for fragment amplification: CCGGTATGCGGCGAAATAC
F, for fusion PCR: GTTGAAGGCAGACGGACTC
R, for fragment amplification: cgaagagggtgaagagcattgTACTCGGTGAGATGAGCAAG

Primers for 3' flank:
F, for fragment amplification: agtgcctcctctcagacagCTGGTCTGTGGAGGACGC
R, for fusion PCR: AATGCATATATTCGCGACCTC
R, for fragment amplification: GAAATAAGATCCCTCTTGCAC
R, for diagnostic PCR: TGTTGACGCGTAATATAGCTC

Primers for flanks to delete AN10028

Primers for 5' flank:
F, for diagnostic PCR: TTGCTTGCGCTGCTGATAAG
F, for fragment amplification: GGACGCCGACTCGATAAT
F, for fusion PCR: AAGACCACAAGGGTCTCAAC
R, for fragment amplification: cgaagagggtgaagagcattgCCTTGGTCCCAGGTATCAG

Primers for 3' flank:

F, for fragment amplification: agtgcctcctctcagacagAATTAGGGCTAGATCGATAGG
R, for fusion PCR: AGTTTGCGCACCTCAAGGC
R, for fragment amplification: AAGAGAAAGCTCGGTGGCG
R, for diagnostic PCR: TTCTGGTCGTGGGACACAG

Primers to amplify fragments for fusion PCR to integrate a wild-type copy of AN10028 at the *riboB* locus

Primers to amplify *riboB* 5' flank:
F, for diagnostic PCR: AGTGTTGGTGGAAGTGCCC
F, for fragment amplification: CCAGAAGCACTGATTGACTC
F, for fusion PCR: CCATTCACGGTTTCCTGGC
R, for fragment amplification: cgaagagggtgaagagcattgAGCGATATACAACCGAATTCC

Primers for *riboB* 3' flank:
F, for fragment amplification: gcatcagtgcctcctctcagacagAGATACGATGGAGATGATTCC
R, for fusion PCR: GGTCGATGTTACCGCAACG
R, for fragment amplification: CAGCAGGGTTGGAGCATAG
R, for diagnostic PCR: CGGGCTGGGGCTATTGTTG

Primers to amplify AN10028:
F primer: caatgctcttcaccctcttcgAAAGAGCTGAGGGAGAAACC
R primer: ttcaagcaaatcaatcccaggCTAATTCACTTCCACCAAGCA

Primers to amplify *AtriboB*:
F primer: CCTGGGATTGATTTGCTTGAA
R primer: ctgtctgagaggaggcactgatgcCCGGACTATGTCGCAGCC

Primers for a fragment to complement genes in the *fwA1* region

To complement AN1087:
F primer: ATGTAGTGAAATGAGCCTGTC
R primer: TGACGCTTGACAGTCTAAAAC

To complement AN1059:
F primer: GAGTGTCCATGGTCCAACG
R primer: GCCTTTAAGCGGTTACTAGG

To complement AN1063:
F primer: CACCAAGCAGGGAGCCAC
R primer: ATAATTCTCCAAGCCGGAAAC

To complement AN10167:

F primer:	ACTATCTCTACAAAACCTCCG
R primer:	AGCAACGTTATACTGGCGAC

Primers for flanks to delete AN1087

Primers for 5' flank:

F, for diagnostic PCR:	AGCGCACGGAGAGTTGCC
F, for fragment amplification:	ATGCTACACTTGTTATGCCAC
F, for fusion PCR:	AATCTATAACTGTCCAGCTCG
R, for fragment amplification:	cgaagagggtgaagagcattgAGGTGATGTGGAAGTGGTTG

Primers for 3' flank:

F, for fragment amplification:	gcatcagtgcctcctctcagacagACGATGATGAATTGCTCGGC
R, for fusion PCR:	TCGTATCCCTCCACGAGAC
R, for fragment amplification:	TTCCACGAACCAGCCCTTC
R, for diagnostic PCR:	ATATCCTTTCTCTACAGCTCG

Primers for flanks to delete the 5' region of AN1087

Primers for 5' flank:

F, for diagnostic PCR:	AGCGCACGGAGAGTTGCC
F, for fragment amplification:	ATGCTACACTTGTTATGCCAC
F, for fusion PCR:	AATCTATAACTGTCCAGCTCG
R, for fragment amplification:	cgaagagggtgaagagcattgAAGGGATGCAACCTTGAACC

Primers for 3' flank:

F, for fragment amplification:	gcatcagtgcctcctctcagacagGTCGGGTTTATTCAAAGTCGT
R, for fusion PCR:	TATCAGCACACAACTACCAG
R, for fragment amplification:	GACTGCTGCCGTTACCAAC
R, for diagnostic PCR:	TCATCCACAACAGCCGTCG

Primers for flanks to delete the 5' region of AN11841

Primers for 5' flank:

F, for diagnostic PCR:	TTCATCTTAGGTCCGCCTAG
F, for fragment amplification:	CTCTCAAACATCCGCTGGC
F, for fusion PCR:	AGCCCTGACAACCTCAACC
R, for fragment amplification:	cgaagagggtgaagagcattgCGATAAAGCTTCGGTTCAATC

Primers for 3’ flank:

F, for fragment amplification:	gcatcagtgcctcctctcagacagGGAGACAAGAGAATCGTAGG
R, for fusion PCR:	GTGGAACGTCAATAGATGGC
R, for fragment amplification:	TCGTCCGCGAAACACTTCG
R, for diagnostic PCR:	ATCCATCATCGGTTGACAATC

Primers to delete AN1088

Primers for 5’ flank:

F, for diagnostic PCR:	TGAGGCTCAAATGCTTGAAC
F, for fragment amplification:	CTGCCAACCCGGCAGATC
F, for fusion PCR:	CGTCCCATTCTGCACGCC
R, for fragment amplification:	cgaagagggtgaagagcattgCTGTACAGGTCAGGGTAGG

Primers for 3’ flank:

F, for fragment amplification:	gcatcagtgcctcctctcagacagGGACAGGCTCATTTCACTAC
R, for fusion PCR:	CGGTACTAACTGGATTCAG
R, for fragment amplification:	ATTGGATCAAGGTATGTCTGC
R, for diagnostic PCR:	ACGTATGACAACATCCAGATC

Primers to repair the promoter of AN1088

Primers for 5’ flank:

F, for diagnostic PCR:	TTCATCTTAGGTCCGCCTAG
F, for fragment amplification:	CTCTCAAACATCCGCTGGC
F, for fusion PCR:	AGCCCTGACAACCTCAACC
R, for fragment amplification:	cgaagagggtgaagagcattgCGATAAAGCTTCGGTTCAATC

Primers for 3’ flank:

F, for fragment amplification:	gcatcagtgcctcctctcagacagAAGGGATGCAACCTTGAACC
R, for fusion PCR:	AGACGGCGTGATTCCGAAG
R, for fragment amplification:	ACGTTCCGAGAGAATGGATC
R, for diagnostic PCR:	GACGTGCCCTTAACACCTG

Primers for amplifying and sequencing the *fwA* region

F Primer:	AGACGGCGTGATTCCGAAG
R Primer:	TGACGCTTGACAGTCTAAAAC

Primers for selectable marker sequences

AtpyrG:

F:	caatgctcttcaccctcttcTGGGCGGGTTCTTTTGGTTT
R:	ctgtctgagaggaggcactACTATCAAGTAGTACGAGTTAC
F, internal, for diagnostic PCR :	CAGAAGCAGTACCATGGCG
R, internal, for diagnostic PCR:	ATGTGCGCCCACTCGGAG

AtriboB:

F:	CCTGGGATTGATTTGCTTGAA
R:	ctgtctgagaggaggcactgatgcCCGGACTATGTCGCAGCC
F, internal, for diagnostic PCR:	AACCAGAGAATCCAGGATATC
R, internal, for diagnostic PCR:	CCGCACCAATGGCGCTTG

AfpyroA:

F:	CAATGCTCTTCACCCTCTTCG
R:	CTGTCTGAGAGGAGGCACTGATGC
F, internal, for diagnostic PCR:	TCAACGCGGAGCAGGTATG
R, internal, for diagnostic PCR:	GATCTCCTTGATCATGCTGG

Primers to delete *yA*

Primers for 5’ flank:

F, for diagnostic PCR:	TCTGTCGTCAGGTGTAGATG
F, for fragment amplification:	GTGCACCATCGACCGTTTG
F, for fusion PCR:	GCCAGATGTAGAATCGGCC
R, for fragment amplification:	cgaagagggtgaagagcattgCAAGGGAGTCTTGTCTGCTC

Primers for 3’ flank:

F, for fragment amplification:	gcatcagtgcctcctctcagacagGGTGAGCTCTCATATTCGTACT
R, for fusion PCR:	GTCGCATCTGTCCTCATGC
R, for fragment amplification and diagnostic PCR:	GTATGCGAGTCTGCAGCAAAG

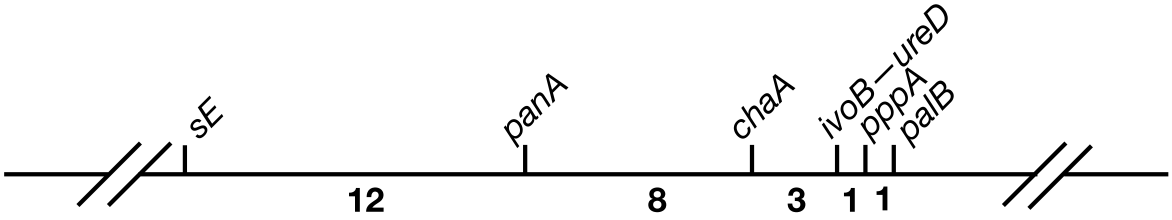

Supplemental Figure S1. A map of the region around *chaA*. The region is on the right arm of chromosome VIII. The numbers below the lines are distances between genes in centimorgans. (Based on Clutterbuck, A.J. The validity of the *Aspergillus nidulans* linkage map. *Fungal Genet Biol*. 1997 *21*, 267-277. doi:10.1006/fgbi.1997.0984.)

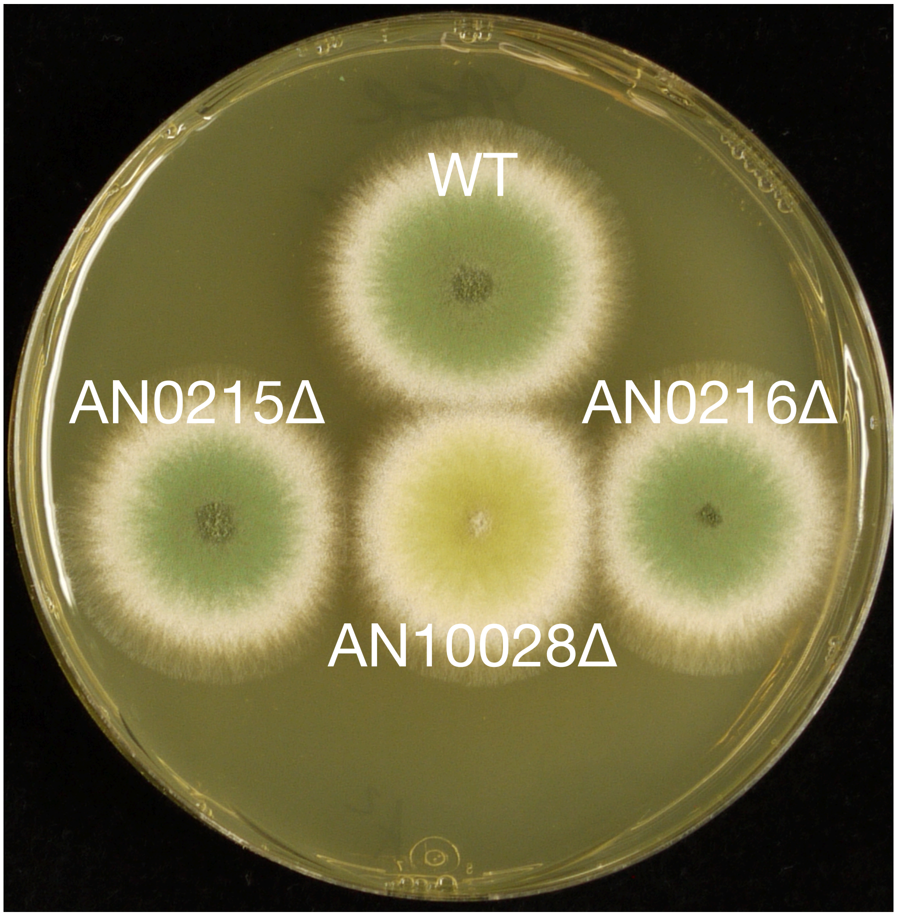

Supplemental Figure S2. Phenotypes of deletions of *chaA* candidate genes. The wild-type strain is LO1. Deletions of AN0215 or AN0216 do not change the spore color, but deletion of AN10028 results in the chartreuse spore color.

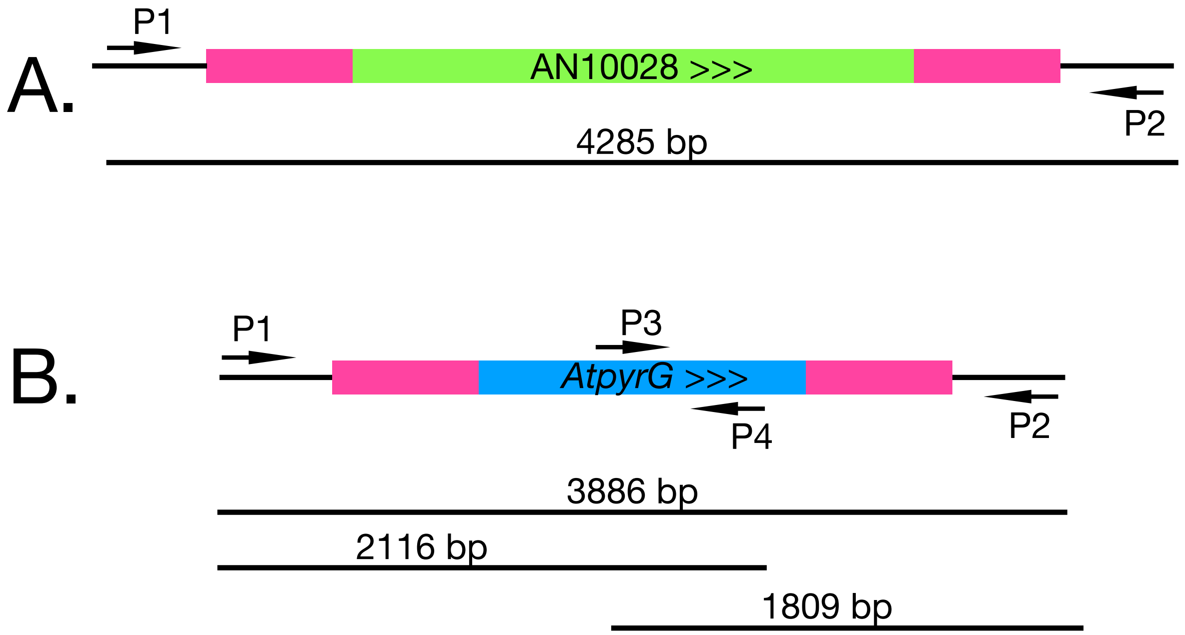

Supplemental Figure S3. Schematic diagram of diagnostic PCR for deletion of *chaA*. A. The wild-type *chaA* locus. Primers P1 and P2 are outside of the fragment used for transformation. Amplification with these primers will yield a fragment of 4285 bp. B. The *chaA* locus replaced by *AtpyrG.* Amplification with primers P1 and P2 is predicted to yield a product 3886 bp in length. Primers P3 and P4 are specific for *AtpyrG*. P1 and P4 are predicted to yield a fragment 2116 bp in length whereas P3 and P2 will yield a fragment 1809 bp. P3 and P4 are not present in AN10028 so if AN10028 has not been replaced, P1 and P4 will not yield a specific product, nor will P3 and P2.

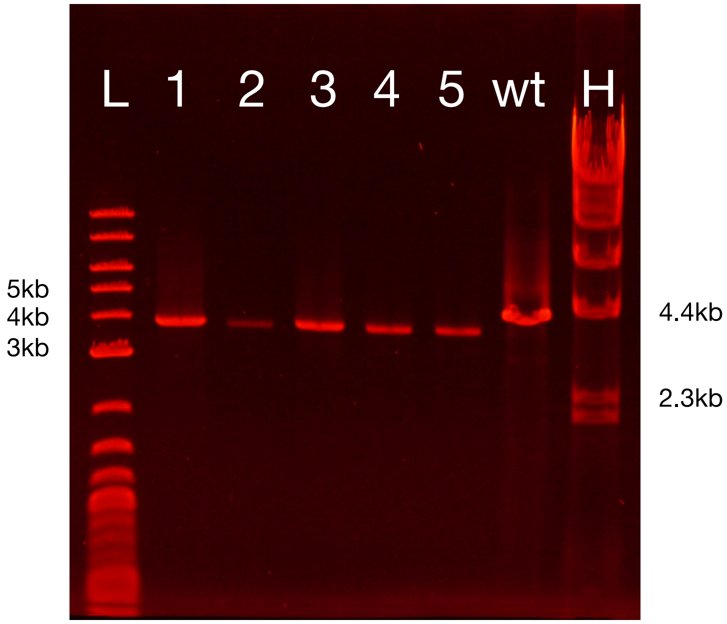

Supplemental Figure S4. Diagnostic PCR of *chaA* deletion transformants using outside primers (P1 and P2 in Supplemental Figure 3). Lane L is a molecular weight marker ladder. Sizes of relevant bands are shown at the left. Lane H is a lambda HindIII digest. Sizes of relevant bands are shown at the right. Lanes 1-5 are diagnostic PCR reactions run on miniprep DNA from five putative *chaA* deletant transformants. The wt lane is an amplification of purified wt DNA (strain LO1). As shown in Supplemental Figure 3, the bands in the transformants are the predicted size for AN10028 replaced with *AtpyrG,* and the band in the wt lane is the size predicted if AN10028 is intact.

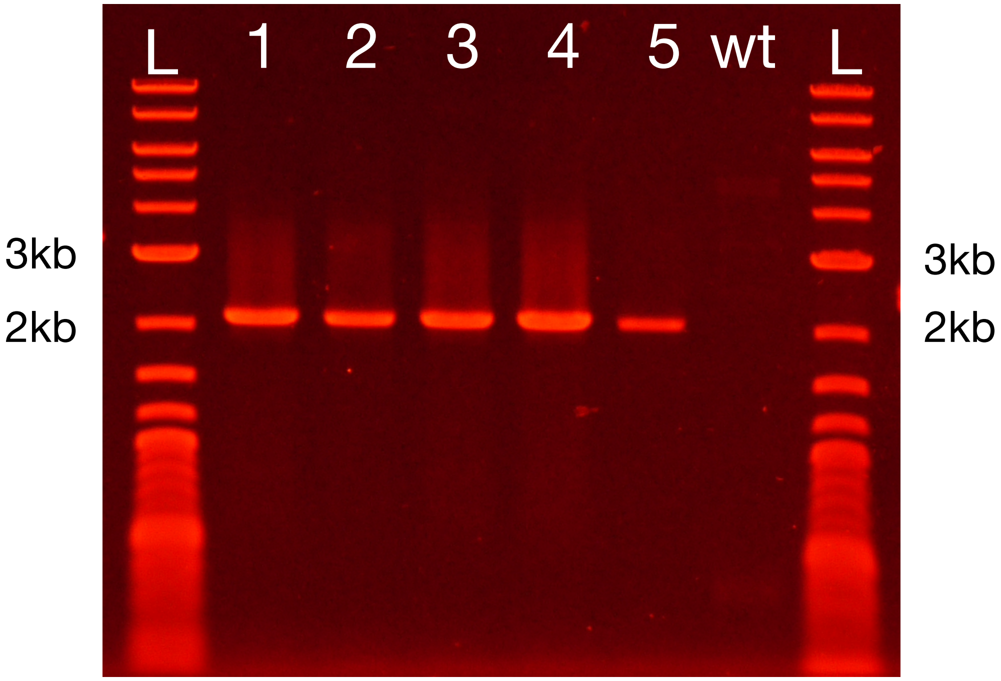

Supplemental Figure S5. Secondary diagnostic PCR of putative deletions of AN10028. Amplification was with primers P1 and P4 in Supplemental Figure 3. In AN10028 deletants, a band of 2116 bp is predicted to be amplified, but P4 does not bind to AN10028, so if AN10028 is present no specific band will be amplified. Lanes marked L contain a molecular weight marker ladder and sizes of relevant bands are shown. Lanes 1-5 are diagnostic PCR reactions run on miniprep DNA from five putative *chaA* deletant transformants (the same samples as in Supplemental Figure 4). A band of the correct size is amplified in each instance. Lane wt is DNA from a wild-type strain (LO1) amplified with the same primers. As expected, there is little or no amplification with DNA from the wt strain.

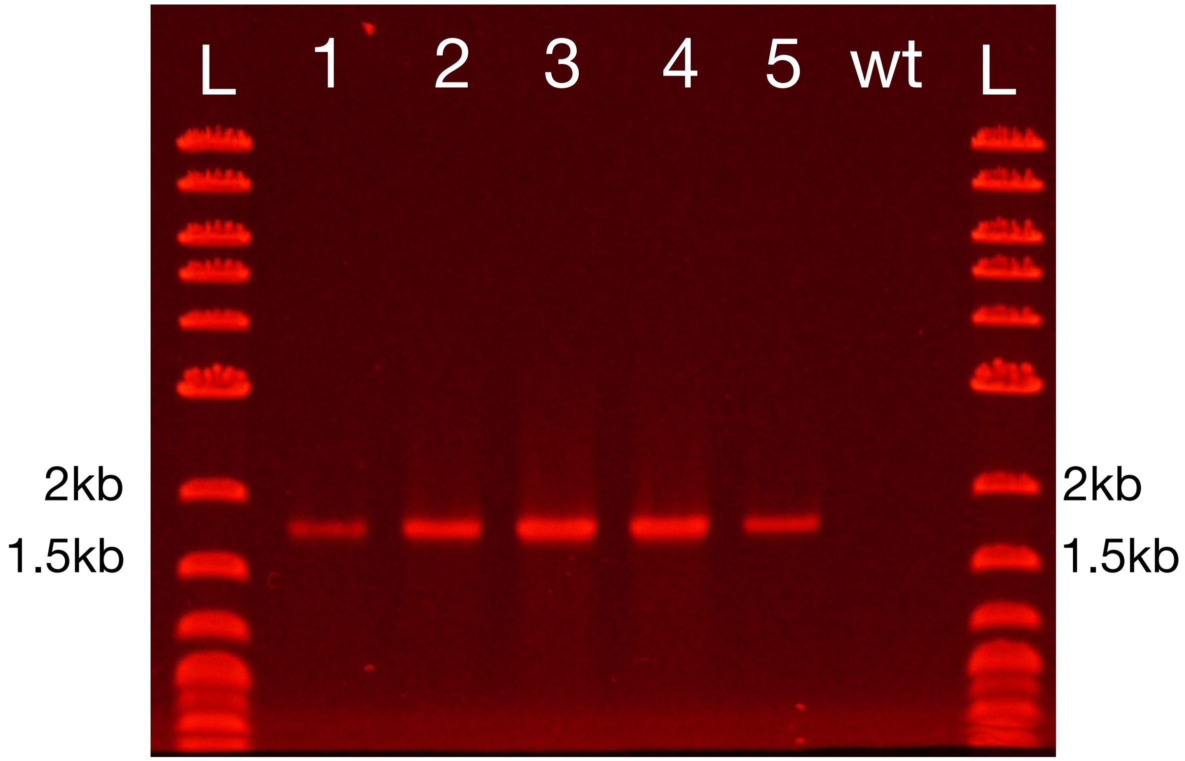

Supplemental Figure S6. Tertiary diagnostic PCR of putative deletions of AN10028. Amplification was with primers P2 and P3 as shown in Supplemental Figure 3. In AN10028 deletants, a band of 1809 bp is predicted to be amplified, but P3 does not bind to AN10028, so if AN10028 is present no specific band will be amplified. Lanes marked L contain a molecular weight marker ladder and sizes of relevant bands are shown. Lanes 1-5 are diagnostic PCR reactions run on miniprep DNA from five putative *chaA* deletant transformants (the same samples as in Supplemental Figures 4 and 5). A band of the correct size is amplified in each instance. The wt lane is DNA from a wild-type strain (LO1) amplified with the same primers. As predicted, there is little or no amplification with DNA from the wt strain.

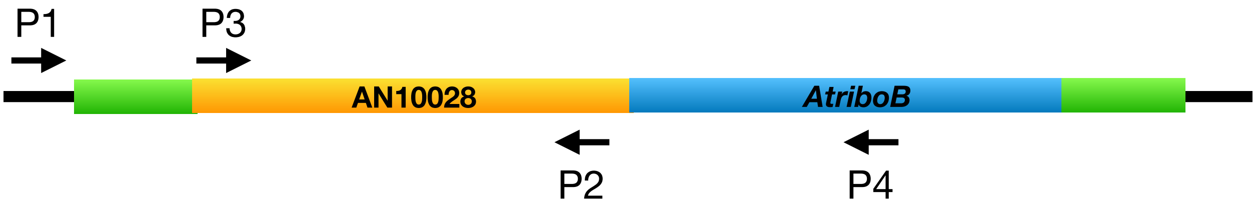

Supplemental Figure S7. Schematic diagram of diagnostic PCR for restoration of *chaA*. Note that AN10028 and *AtriboB* are inserted in place of *riboB2* (*s*ee Figure 4 in the text). Primer P1 is in the region flanking *riboB*. If AN10028 is inserted correctly (as shown), amplification with primers P1 and P2 is predicted to give a band of 3.3 kb. Since AN10028 is distant from *riboB* in the wild- type genome, amplification of wt DNA with P1 and P2 will give no specific band. Amplification with prime rs P3 and P4 is predicted to give a band of 3.5 kb. Since *AtriboB* is absent from the *A. nidulans* wt genome, amplifications with P3 and P4 will give no specific band.

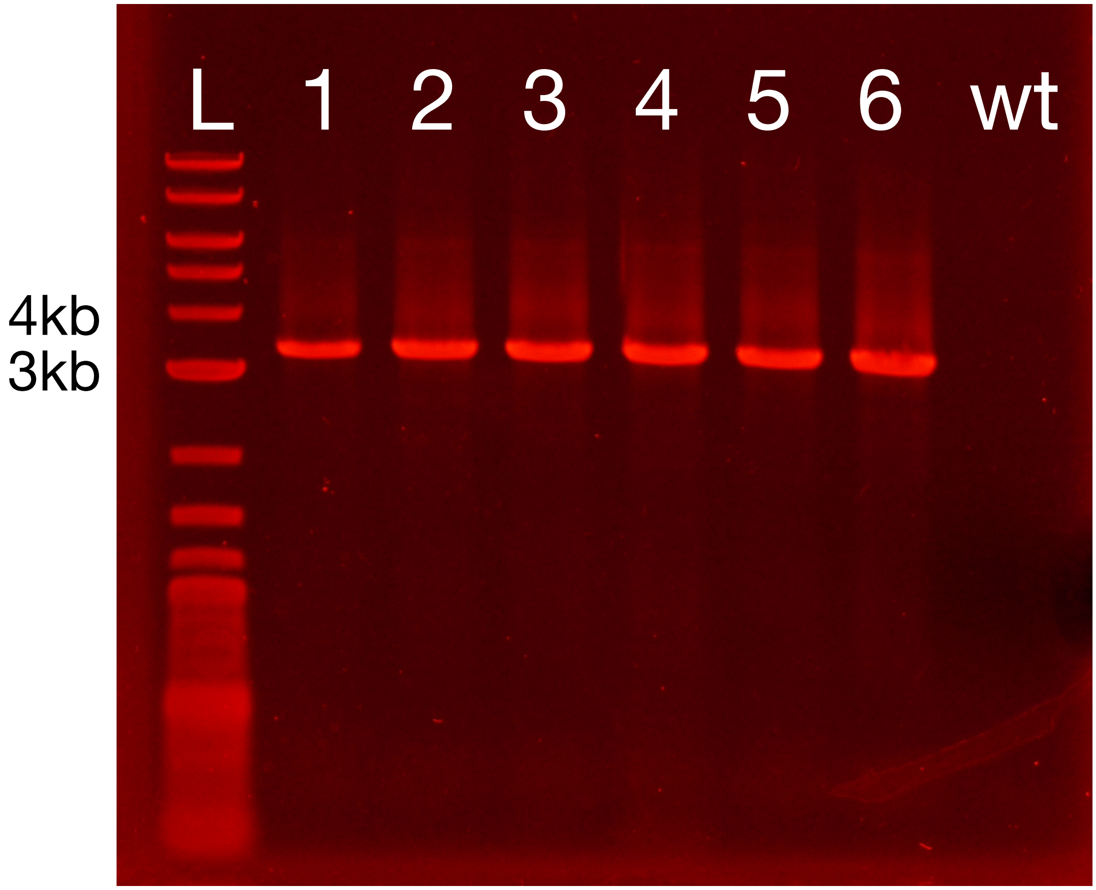

Supplemental Figure S8. Primary PCR verification of the restoration of *chaA*. Amplification was with primers P1 and P2 as shown in Supplemental Figure 7. The lane marked L contains a molecular weight marker ladder and sizes of relevant bands are shown. Lanes 1-6 are amplifications of DNA minipreps from transformants in which AN10028 (putative *chaA*) has been restored (see Figure 4 in the main text). All give the expected band of 3.3 kb. As anticipated (see Supplemental Figure 7), there was no amplification of wt DNA.

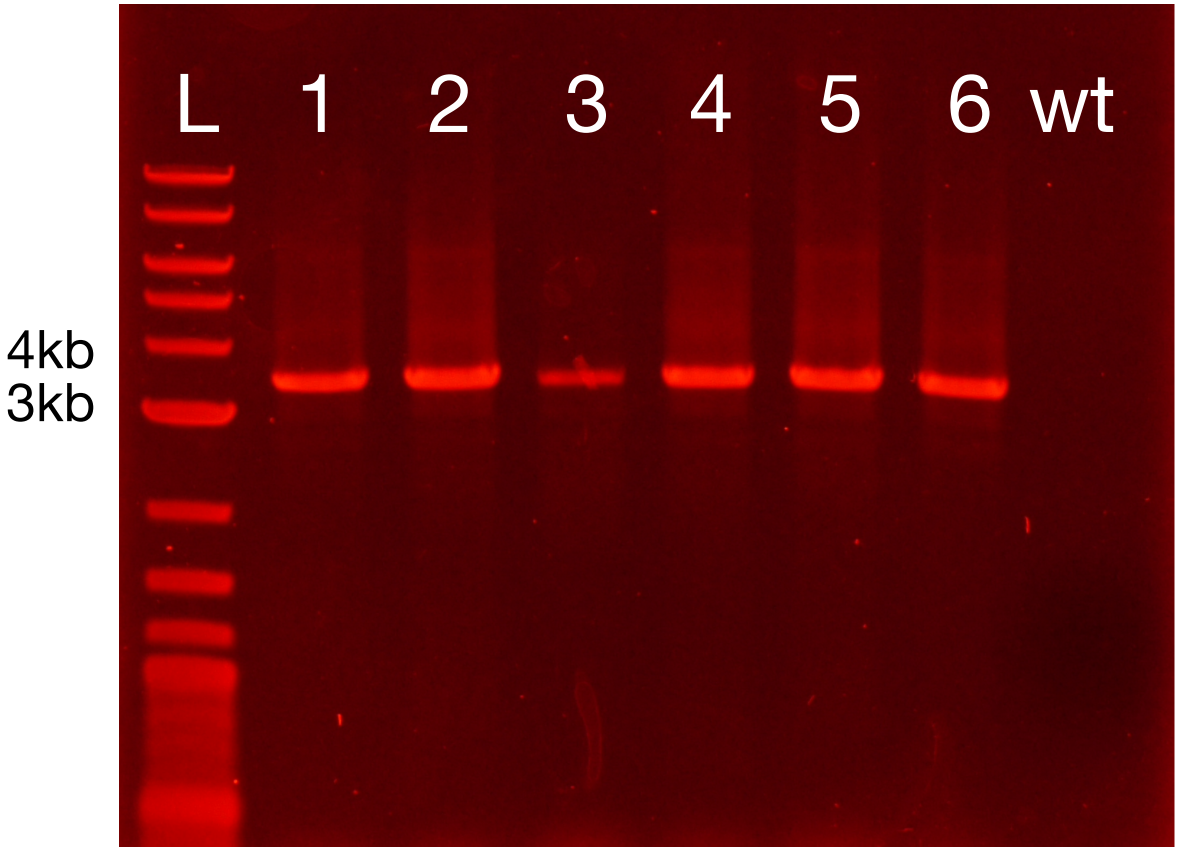

Supplemental Figure S9. Secondary PCR verification of the restoration of *chaA*. Amplification was with primers P3 and P4 as shown in Supplementary Figure 7. The lane mark ed L contains a molecular weight marker ladder and sizes of relevant bands are shown. Lanes 1-6 are amplifications of DNA minipreps from transformants in which AN10028 (putative *chaA*) has been restored (see Figure 4 in the main text). All give the expected band of 3.5 kb. As anticipated (see Supplemental Figure 7), there was no amplification of wt DNA.

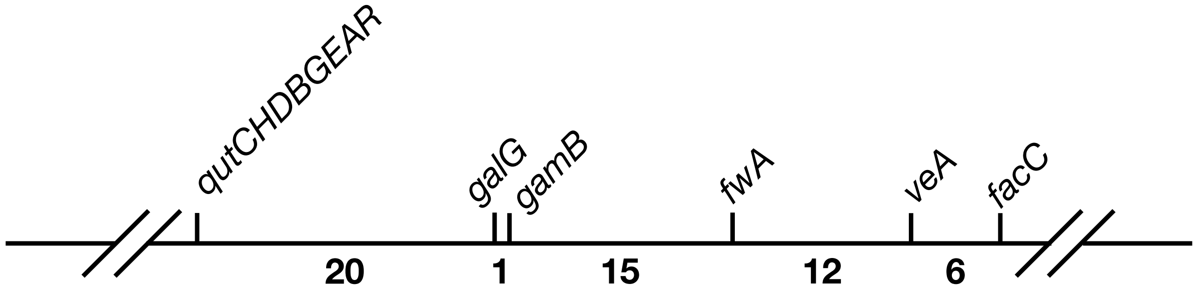

Supplemental Figure S10. A genetic map of the *fwA* region of linkage group VIII. Numbers below the line are distances between genes (centimorgans). qutCHDBGEAR is a complex of genes that map together, including *qutD*. *qutD* has been determin ed to be AN1138. Note that while *galG* and *gamB* are nearer to *fwA* than *qutD*, the genes corresponding to these genetic loci have not been determined. (Based on Clutterbuck, A.J. The validity of the *Aspergillus nidulans* linkage map. *Fungal Genet Biol*. 1997 *21*, 267-277. doi:10.1006/fgbi.1997.0984.)

Supplemental Figure S11. The *fwA* region containing three genes, AN1088 (*fwA*), AN1087 and AN11841. The sequence of a single DNA strand is shown. Introns are not marked. Start codons are highlighted in green and stop codons in red. The *fwA1* deletion is shown in bold italic text.

TTTCCGTCAAAGCGCACGGAGAGTTGCCATACACTATTGTTATGCTACACTTTATGCTACACTTGTTATGCCACACGTTT

CTTTTTGTGATTCCCTGGGTTGCATAGGTTTCATTGATTAAGGTTCGAAAATTAGGCGCAGGGGGGAGGCGGTTTGTTAC

TCAGCATTATATATAAATCTATCTCATCGAAGTATCAACCAATCTATAACTGTCCAGCTCGATCCAGCCCTCTAGCATTA

AN1088

AACATTCTTTATGGTCAAAATGTAGTGAAATGAGCCTGTCCTCAATTTTTCAACTGCCCCAACCCTTCCTCCGGCGAGAT

GAATCTCGTCCTGTACCCAATCGATATCCTATTACCCCCCAAACTCATCAGCACACTTTTGCCTTGAAATATAGGAAAGA

AGGACTAACCCAGACTTGCTCTCGTCCGCAAAGTTCACATGATCCGGCTTAACAGTTTTCCGGAACTCCTCGTCCTCCCA

GAGTCTTGTAAACTGCCTAATATCATCCATAACAAACTGCACGATGTAGTCACAATCCGCTAGCTTCTCGGTCATATGCA

TCTGCTCGACGTGGGGTGTGAAGGTGGAGAATTGGTCGATCTGCACATATCCAGTCAATAATCCACTTCCCTTAAAGAGT

TTTGGGAGGATATATGAAGTAGAAGTACCAGGCTATACTCCTTAACACCGTATTTCAGCAGGAAGGGTAGCGCAAGGGGT

GCATGGTGGTTTGTTAAATACTCGTGCAAGGAGGACTCATCGAGACCAGGCTTGCGGAAGCCAGTTATTGTTAAGACCAC

AN1088

TTCGGTGGGCATTCTGTACAGGTCAGGGTAGGTCTGCTATTCCTTGAGGCGGAAGTGGGCGATTGAATTGAAGTTACTGC

Deletion

ACTTGAAGGGAAGGTGCAGTAGTAAGGGTTGAATATTGGCAGTTCAAGTCTCTATATATCTCGAAGTGATCT***TTGGCTAG***

AATGCCGGATGCTACATAGGTCAAGCTAATGTTGCAGTATACTTAATCCGGACAGAGTATTGCTTTTTCGACGGAAACGT

CTGACTAGTCTACTTTGAAACTTAGACAAATAAGCTGTATAATGAGAGTTCATGTTGTACAGCGCAGTTCTCGATCCTAA

TTTAAGGTTCAAGGTTGCATCCCTTGACTGCCCAGTCCATTCTCACTACCACCTGGGCAACCACTTCCACATCACCTACC

AN1087

TCACGATCGATCCATGGTTCAATTACTGACTGATTCCACAATGTTCACGCACACAGCCATCTTTACCCTCTCTTTTCTAC

TTACAGTCTCTTTCCTTCTGTTTATATATCTCATCCACCCGCTCTTCCTCTCCCGCCTCTCAAAGCTCAACATACCCAAT

GCGCACTTCACCTCCCCGCTGTCCAGCTACTGGATTAACTCCATCCGGCATGCCGGATTAGAAACAGTCACGATTCAAGC

TCTGCATGCAAAACATGGGCCTGTCGTTCGATTATCACCGAACGAACTGAGTGTCAACTCTCTCCATGGACTACGGGTGA

TCTATACTGGAGCGTTCGAGAAACACGCTCTGTACAGAGACCTGTTTCTCAACTTCCATACGGAGAATCTGGTTGGGATG

CTGGGTAATAAAGAGCATGCGCGGCAGAAGAGAATGCTTAGTCGGGTTTATTCAAAGTCGTATTTACAGGAAAGCGAGGA

TATGAGAGTGATCTCGGCGGTGATTCTATCGACTCGACTCCTACCAATCCTGCAGCGGATCGCGGAAAATGGAGAGACTG

TGAATGTCCTGCCGCTGTTCCAGGCTGTTGGCATGGATTTCACTTCGGCTTACTTGCTCGGCGTGCAGAATGGGACGAGC

TTTTTGTTTGATCTGCCGGGTTGGCAGAGGTGGCTAGAAGAGTATGAAAAGTTCAAGCATTTGAGCCTCAACGAGCGGGC

TGATGGGTTTATTGAACGATGGTGTCTGGATTTATGTCGACAAACGCAAACTGAGTCGAGTAGTAGTGACGCTATATCCA

CCAAACCGGTCGTATACAATGCCCTCCGGCATGGTCTGGAGAAGTCCCCAGATTCCCGTCCCAGCGACCTCGCTATTGCG

TCGGAGCTTCTCGACCACCTTATAGCCGGTCATGAAACGTCGGGGATCACATTCACGTATATGATGTGGGAGCTCTCGCA

TTGTCCAAACCTGCAAGATGAGCTCCGAGCAGAACTGCTCCTACTGCAACCTTCACTAAAGTACCCCTTTCCCTCAGGCG

GTGGAAATGGTAGTTTCCCACATCCATCATCGGTTGACAATCTTCCCCTCCTTGATGCGATCGTCCGCGAAACACTTCGG

CTCCATGCCCCCGCCTCATCACCACTACCCCGTGTAACTCCAGATACGCCAGGTGGAACGTCAATAGATGGCTTCGATGG

TATTCCAGGCGGCATCACTGTCAGCTCATCTGCATATACCCTCCATCGGATTGAGGAGGTATATCCGCAGCCAACGGAGT

GGCTACCACAGCGATGGCTCGATCCTGGGTCTGGAAAGAAACATGATATGCGGCGGCTCTGGTGGCCGTTTGGGAGTGGA

GGTAGGATGTGTCTAGGGAGCAATTTTGCGCTGCAGGGTACTTCCCCAATATTTTCTTTCAGTTAATACTGTTTCTTGTC

TTCTTTGTCTTGGTAGTTGTGTGCTGATAGGATACAGAAATCAAGTTGGTAACGGCAGCAGTCTATACCAACTACACGAC

GGCTGTTGTGGATGATGAGGGTATTGAGCAGGACCTCGCAGACTTTATCTCCCTGCCGAAGGGACGCAAGCTGGTGCTGA

AN1087 AN11841

GGTTTGATCCTATTAACAGGGCTTAACAGCATGGCACGTGTCTGCTCACGATGATGAATTGCTCGGCTCACGCACCATAC

AATAAAGCTGCAACAGTCAGCCATTGCACTCGTATAGAGAAGAGGAGCACTTACTTTACAGGCCTCTCCCTCTCCATCAC

CATCCCGACACTCCATCTCCATTCTCAACTGAAACCCAAGTCGCTGATAAATTCCCACATTTGGCTCGAGTCTTGAGCTT

TCTAGATAACACTTCATCCCTTCTCTATCCGCAATTTTAGTAACTTCCTCGAAGAGCTTTCTTCCCACGCCCCTCCCCTG

Deletion

CGCATCGGGCCTGACGGCAACAATATTGCAGAAATAGTACCCCCGTTCATCGTCCCATATAGCTTTCTGGCATTCAGCTT

GCCGCTCTTTCCAGAGGTAGTATCGACGGATATTGAGCCCGCCCCGTCCACCGTGTCGGAGATTATTTAGCCCCTGCCGG

AACCAGAGGAGCCAGGATTGGGACCAGCTGTACCATGATTCTGGTTTTGATAGGGGATGGGGTGCAAGCCAGCATGACAC

GCCTACGATTCTCTTGTCTCCTGCAGCGGAATCTAGTTCTGCACCTGTGTCCACGGCAACATGGAAAATGGCGTTATTGA

TTCCCCAGAGACACCGTGCTTCTAATGAGCCGTAGTTGCGAACTTTGTTGAACTGTTTTCTATGTTAGCTTTCCCCCTCC

TTCTCACAATTCGCGTTTCCAGGTTATATACAAGGAACTGATGATATAAGCATGCACCATTCAATATATACACTGGCGTA

TAACGAGAGCAACAAGCCAGGCCTATGTAGAGAAAAGACAAGAGGGGAGGTGACCATAAGATACATACATTCTGCTTATC

AAATACCCACTGAAAATAGGGATCATCTGCAAAAGCCTGCTGGATCACCTCGATTGCGCCGGGGATCTCGGCTGTCGTGA

AN11841

GTGGGTGCACCTCGATTGGCATATTTCTCTGAGTTTCTTCGTTTTAGACTGTCAAGCGTCAGTATATGGTCTCGTGGAGG

GATACGACGGCGGAAATGCAGTACCTTATGTATAGTACTCTGAAGGGCTGGTTCGTGGAAGTGGATGGCAGGAGTAGATA

CAAAGCAGAATATACAAAGTATTGGTTGATAGTTCCACTTCGAGCTGTAGAGAAAGGATATGGTCAGAGGTCGGTCTGTC

TCGAGAAGAATGATTGTGATAGATTGTGCGTGTATCAAGCAGAGAAGAGGTCTGGTTGGAGATCTTTATTAGTGATAAAC

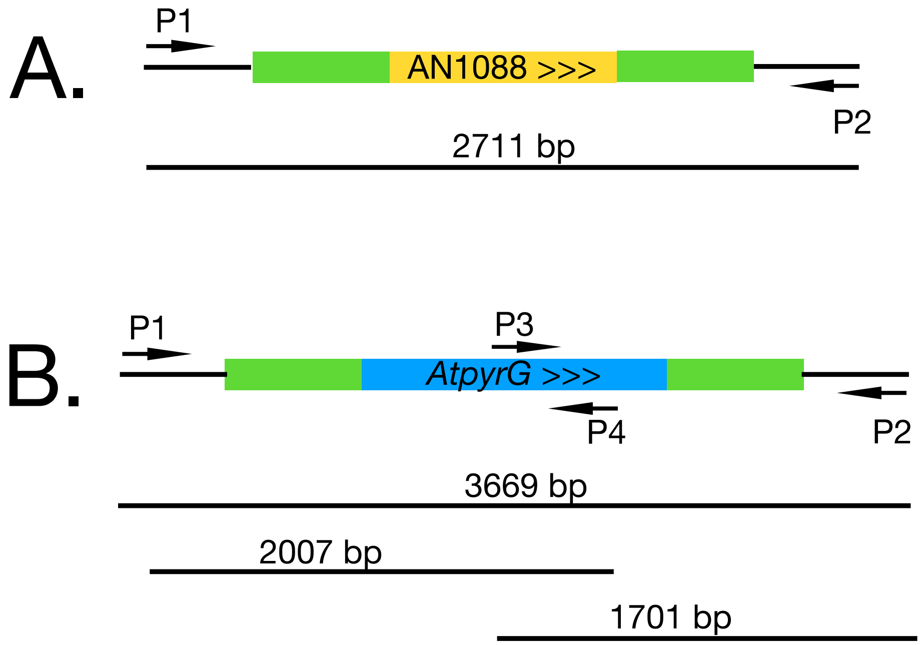

Supplemental Figure S12. Schematic diagram of diagnostic PCR to verify the deletion of *fwA.* Panel A shows the wild type and B shows the replacement of AN1088 with *AtpyrG*. In the wild type, primers P1 and P2 will amplify a band of 2711 bp, whereas in the strain in which AN1088 is replaced with *AtpyrG* the amplified band will be 3669 bp. *AtpyrG* is not present in wild-type *A. nidulans*, so primers P3 and P4 will not anneal and there will be no specific amplification with primer pairs that include P3 or P4. In gene replacement strains (B), however, P1 and P4 will amplify a 2007 bp fragment and P2 and P3 will amplify a 1701 bp fragment.

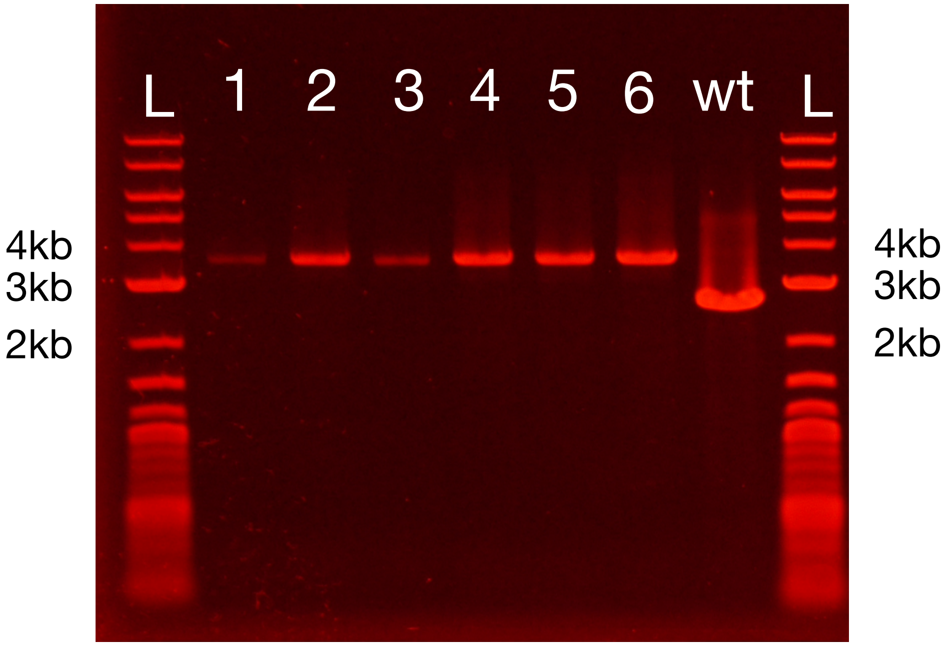

Supplemental Figure S13. Primary PCR verification of the deletion of *fwA* (AN1088). Amplification was with primers P1 and P2 as shown in Supplemental Figure 12. The lane marked L contains a molecular weight marker ladder and sizes of relevant bands are shown. Lanes 1-6 are amplifications of DNA minipreps from transformants in which AN1088 (putative *fwA*) has been deleted. All give the a band of the predicted size (3669 bp) as shown in Supplemental Figure 12. Also consistent with Supplemental Figure 12, amplification of wt (LO1) DNA gave a band of the predicted size (2711 bp).

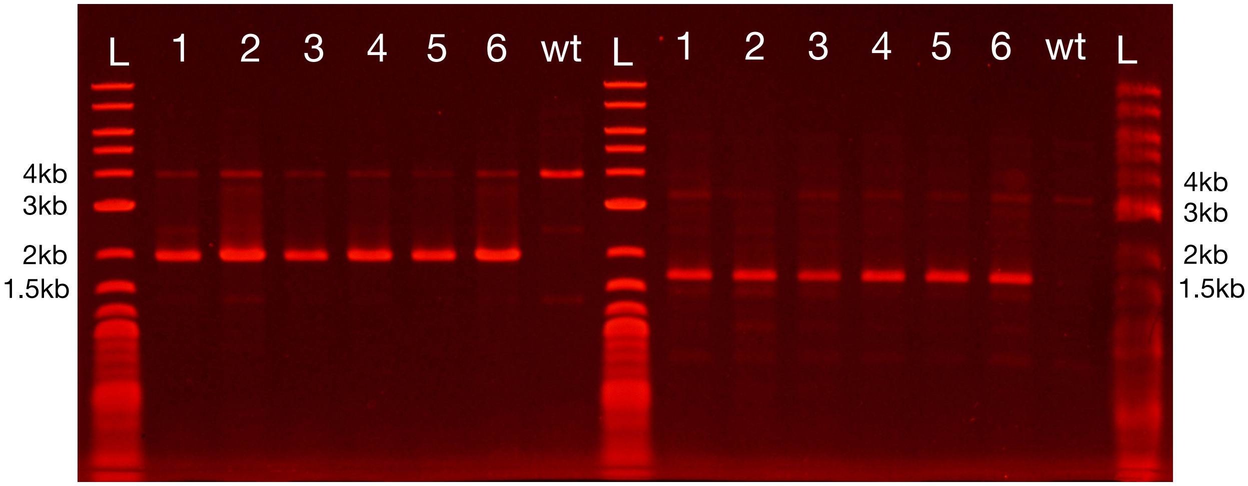

Supplemental Figure S14. Secondary and tertiary verifications of AN1088 (putative *fwA*) deletions. Strains on the left side of the gel and the right side of the gel are the same and they are the same strains used for Supplemental Figure 13. The amplifications at the left side of the gel are with primers P1 and P4 as shown in Supplemental Figure 12. The main band amplified is of the predicted size (2007 bp). There is a 4 kb band in the wt and all the transformant strains, presumably due off-target binding of this primer pair. At the right, amplification is with primers P2 and P3. A band of the predicted size (1701 bp) is amplified in the transformants and, as predicted, no specific amplification is observed with wt DNA.

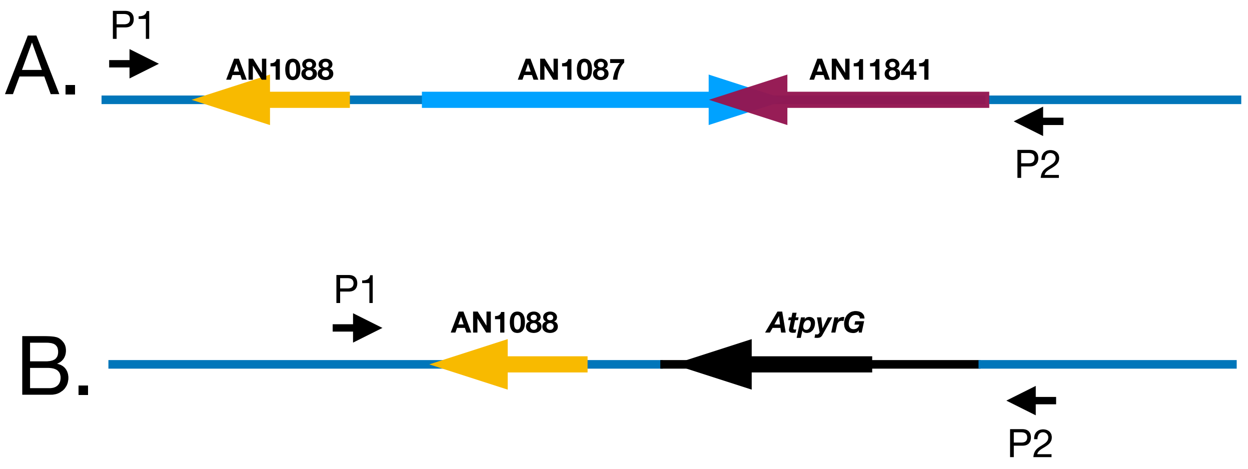

Supplemental Figure S15. Schematic of diagnostic PCR of AN1088 promoter restoration. A. The wild-type AN1088 region. B. The AN1088 region in which the promoter of AN1088 has been repaired and AN1087 and AN11841 have been deleted. (See Figure 8 in the text.) Amplification with primers P1 and P2 is predicted to give a band of 5333 bp with wt DNA and 3891 bp with DNA from transformants with the correctly repaired AN1088 promoter.

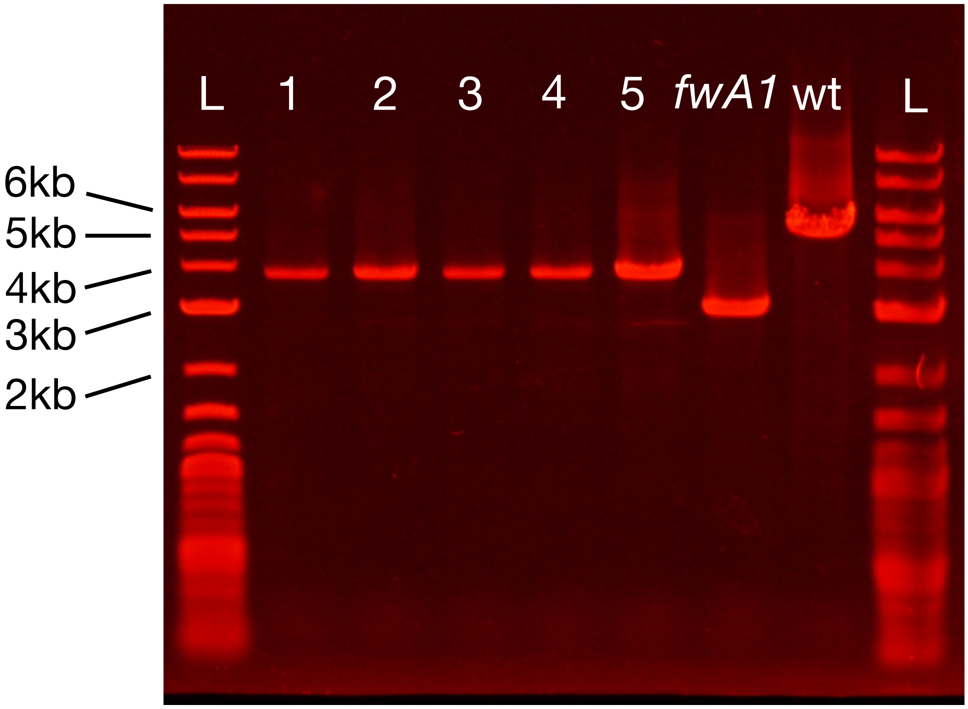

Supplemental Figure S16. Diagnostic PCR of AN1088 promoter repair transformants. Lanes 1-5 are PCR amplifications with primers P1 and P2 (as shown in Supplemental Figure 15) of DNA minipreps from transformants in which the promoter of AN1088 has been putatively repaired. They all contain a single band of the predicted size (3891 bp). Amplification of DNA from a wt strain also gives a band of the predicted size (5333 bp). Because the original *fwA* mutation (*fwA1*) is a deletion, the amplification of DNA from a *fwA1* strain is predicted to result in a band smaller than the band from wt DNA by 2241 bp. This image is of the right half of a gel and the image in Supplemental Figure 13 is from the left half. The left molecular weight ladder on this image is the right molecular weight ladder in Supplemental Figure 13. Additional diagnostic PCR reactions (not shown) were carried out to confirm the promoter repair.
